# Upregulation of adenosine A_2A_ receptor in astrocytes is sufficient to trigger hippocampal multicellular dysfunctions and memory deficits

**DOI:** 10.1038/s41380-025-03115-9

**Published:** 2025-07-23

**Authors:** Agathe Launay, Kevin Carvalho, Athénais Genin, Thibaut Gauvrit, Paola Nobili, Victoria Gomez-Murcia, Emma Augustin, Anaëlle Burgard, Johanne Gambi, Déborah Fourmy, Bryan Thiroux, Didier Vieau, Alexis-Pierre Bemelmans, Stephanie Le Gras, Luc Buée, Miranda E. Orr, Etienne Audinat, Anne-Laurence Boutillier, Gilles Bonvento, Karine Cambon, Emilie Faivre, David Blum

**Affiliations:** 1 University of Lille, Inserm, CHU Lille, UMR-S1172 Lille Neuroscience & Cognition (LilNCog), Lille, France; 2 Alzheimer and Tauopathies, LabEx DISTALZ, Lille, France; 3 Institute of Functional Genomics, University of Montpellier, CNRS, INSERM, Montpellier, France; 4 Université Paris-Saclay, CEA, CNRS, MIRCen, Laboratoire des Maladies Neurodégénératives, Fontenay-aux-Roses, France; 5 Université de Strasbourg, UMR-7364 CNRS, Laboratoire de Neurosciences Cognitives et Adaptatives (LNCA), Strasbourg, France; 6 CNRS, Inserm, UMR-7104, GenomEast Platform, Institut de Génétique et de Biologie Moléculaire et Cellulaire (IGBMC), Université de Strasbourg, F-67400 Illkirch, France; 7 Department of Neurology, Washington University School of Medicine, St Louis, MO 63110 USA; 8 St Louis VA Medical Center, St Louis, MO 63106 USA; 9 Université Paris-Saclay, CNRS, Institut des Neurosciences Paris-Saclay, Saclay, France

**Keywords:** Neuroscience, Diseases

## Abstract

Adenosine is an ubiquitous neuromodulator that ensures cerebral homeostasis. It exerts numerous functions through the activation of G-protein-coupled adenosine receptors (ARs), in particular A_1_ (A_1_R) and A_2A_ (A_2A_R) receptors. Interestingly, A_2A_R levels are upregulated in cortical and hippocampal regions in several pathological conditions such as Alzheimer’s disease, tauopathies or epilepsia. Such abnormal upregulations have been particularly reported in astrocytes, glial cells that play a key role in regulating synaptic plasticity. However, the overall impact and the underlying mechanisms associated with increased A_2A_R in astrocytes remain poorly understood. In the present study, we induced the upregulation of A_2A_R in hippocampal astrocytes using dedicated AAVs and comprehensively evaluated the functional consequences in 4 months-old C57Bl6/J mice. Our results show that A_2A_R upregulation primarily promotes alterations of astrocyte reactivity, morphology and transcriptome, with a link to aging-like phenotype as well as secondary impairments of neuronal excitability and microglial phenotype. These changes driven by a restricted A_2A_R upregulation in hippocampal astrocytes were sufficient to induce impairments of short-term spatial memory and spatial learning. This study highlights the impact of astrocytic A_2A_R upregulation, as seen in various neurological conditions, on the development of a detrimental multicellular response associated with memory alterations and provides an additional proof-of-concept for the value of targeting this receptor in different neurodegenerative conditions.

## Introduction

Adenosine is a widespread modulator of the central nervous system (CNS) involved in a large number of key processes such as of neuromodulation and synaptic plasticity, controlling, among others, cognition and the sleep-wake cycle [1]. The multiple effects of adenosine in the brain are mediated by the activation of adenosine receptors (ARs), in particular A_1_ (A_1_R) and A_2A_ (A_2A_R) receptors. These are G-protein coupled receptors, expressed by neurons and glial cells, that respectively associate to Gi (inhibitory) and Gs (excitatory) proteins, which both modulate the activity of adenylate cyclase and the production of cyclic AMP in an opposite manner. Together, they fine tune synaptic plasticity by regulating neurotransmitter release and uptake (e.g. glutamate and GABA) as well as the function of many other receptors, for instance D2R, mGluR5 or NMDAR [1–9]. This likely explains the large modulatory impact of caffeine, a non-selective AR antagonist, towards brain network activity [1, 10–14].

Parenchymal expression of ARs, especially the A_2A_R subtype, is upregulated in several neurological conditions [15], and particularly in the brain of patients with cognitive pathologies such as Alzheimer’s disease, other tauopathies, depression or seizures as well as related experimental models [16–23]. Accrediting a detrimental role of such A_2A_R dysregulation, receptor overactivation using a selective agonist [24] or its selective upregulation in neurons [16, 17, 25–27] have been shown to favor the development of cognitive impairments. In line with this, normalizing A_2A_R function was found to alleviate behavioral and plasticity impairments in various neurodegenerative models [17, 19, 21, 28–31].

Besides neurons, pathological upregulation of A_2A_R also occurs in astrocytes [18, 22, 32–34], glial cells highly intertwined with synaptic processes [35]. Few in vitro studies demonstrated that A_2A_R upregulation in astrocytes functionally alters several of their known functions such as the recycling of glutamate, the Cx43 gap junction hemichannels and their pro-inflammatory profile [6, 7, 36–38]. In vivo, effects of such A_2A_R astrocytic dysregulation remain largely ill-defined. To fill this gap, the present study aimed at investigating the functional outcomes of A_2A_R astrocytic upregulation on hippocampal astrocyte biology as well as its consequences on neuronal and microglial biology and, ultimately, memory.

## Materials and methods

All methods were performed in accordance with the relevant guidelines and regulations.

### Animals

Two-months male C57Bl6/J mice (from Charles Rivers) were maintained in groups of 5-6 in ventilated cages in a SOPF facility (12 h/12 h light/dark cycle, 22 °C), with *ad libitum* access to food and water. The animals were maintained in compliance with European standards for the care and use of laboratory animals and experimental protocols were approved by the CEEA75 ethical committee (12787-2015101320441671v9). The sample size was chosen based on preliminary experiments in order to comply to the 3R rules.

### Astrocytic A_2A_R hippocampal overexpression

**Fig. 1:**
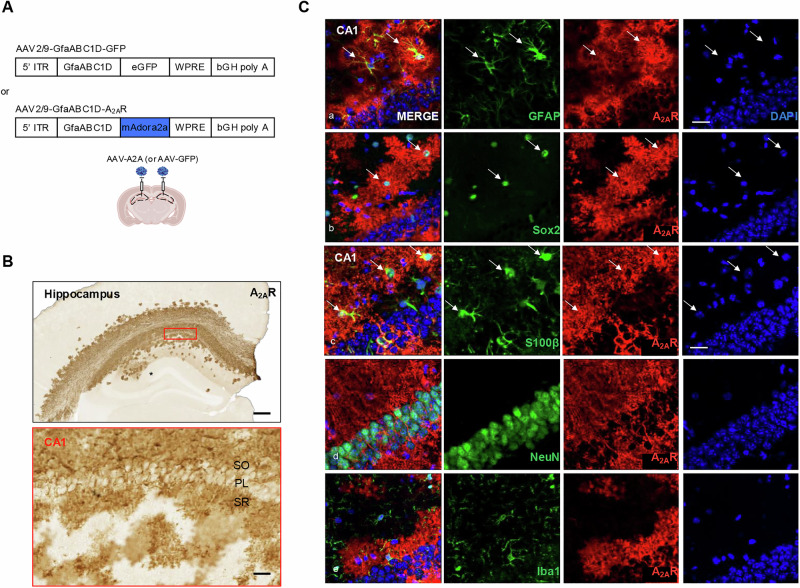
AAV-based astrocytic A_2A_R upregulation in the CA1 hippocampus in mice. **A** Astrocytic A_2A_R upregulation (or the GFP as control) has been obtained following bilateral hippocampal injection (CA1) of AAV2/9 carrying a transgenic murine A_2A_R (AAV-A2A; blue) or enhanced form of GFP (AAV-GFP; white) genes under the control of the GfaABC1D astrocytic promoter. **B** Representative images of A_2A_R immunostaining highlighting A_2A_R upregulation in the mouse CA1 hippocampus (scale bar=500 μm, upper picture) and, at higher magnification, the distribution within the *stratum oriens* (SO), pyramidal layer (PL) and *stratum radiatum* (SR) sublayers of CA1 (scale bar=60 μm, lower picture). **C** Representative images of A_2A_R co-immunostainings with the astrocytic markers GFAP (a), Sox2 (b) and S100β (c), neuronal marker NeuN (d) and microglial marker Iba1 (e) highlighting the exclusive expression of A_2A_R in CA1 astrocytes (scale bar=20 μm).

Upregulation of the murine A_2A_R (mAdora2a), or the enhanced form of the green fluorescence protein (eGFP) as control, were obtained thanks to an adeno-associated virus (AAV) of 2/9 serotype with gene expressions under the control of a GfaABC1D astrocyte-selective promoter (AAV2/9-GfaABC1D-A2A or AAV-A2A; AAV2/9-GfaABC1D-GFP or AAV-GFP, respectively) (Fig. 1; Supplementary Figure 1). WT animals were randomly ascribed to the GFP or the A2A groups. For the surgical procedure, mice were deeply anesthetised with ketamine (150 mg/kg) and xylazine (10 mg/kg). Buprenorphine (0.05 mg/kg) was injected subcutaneously 30 min before the beginning of surgery followed by lidocaine (5 mg/kg) applied on the scalp. The AAVs were bilaterally injected in the CA1 hippocampal area of two months old C57Bl6/J mice at the following stereotaxic coordinates: -2.5 mm rostro-caudal; +/-1.5 mm medio-lateral axis; -1.7 mm dorso-ventral) with a final concentration of 1.10^9^ vg/μl and a total volume of 2 μl per injection site, at a rate of 0.25 μl/min. The needle was left in place for 1 min before the viral vector injection and remained in place for 5 additional minutes following injection, before being slowly removed. The skin was sutured and mice were allowed to recover. Except for behavioral studies where animals were investigated at the age of 3 to 4 months (i.e., one to two months post-injection), all other evaluations were performed at 4 months of age (i.e., two months post-injection). Noteworthy, in these conditions, AAV-based expression of GFP in astrocytes elicited neither astrocytic nor microglial reactivity, respectively assessed by GFAP and Iba1 immunostainings, as compared to PBS (Phosphate-buffered saline) injection (not shown).

### Behavioral evaluations

Behavioral experiments as detailed in Supplementary methods were conducted on animals randomly assigned by experimenters blinded to the injection group.

### Euthanasia

Mice were sacrificed two months post-AAVs injection. They were deeply anesthetized with dolethal (200 mg/kg, intraperitoneal injection, i.p) and lidocaine was applied on the skin and ribs before transcardiac perfusion with cold NaCl (0.9%). Brains were removed and post-fixed in 4% paraformaldehyde (in PBS; pH 7.4) for 24 h at 4 °C and transferred into sucrose 30% overnight for saturation before being frozen. Coronal brains sections (35 μm) were cut using a Leica cryostat and stored in a PBS-azide (0.2%) at 4 °C until immunohistochemical analysis (A_2A_R, GFP, Iba1, GFAP, STAT-3, YAP, S100β, Sox2, NeuN, CD68, HMGB1). Immunohistological and quantification procedures related to these samples are described in the Supplementary methods.

### Transcriptomic analysis of hippocampal astrocytes

#### Tissue preparation

The cell sorting method was performed using the Neural Tissue Dissociation Kit (#130-092-628; Miltenyi Biotec; Supplementary Figure 2A). To do so, left and right hippocampi were dissected out following cervical dislocation and directly collected in Hank’s balanced salt buffer (HBSS 1X; #55021 C; Sigma) and kept at 4 °C. Hippocampi were then cut using dissecting scissors and incubated for 15 min at 37 °C to facilitate enzymatic digestion. Additional cell dissociation was performed using polished Pasteur pipettes with three different diameters (from the largest 5-6 mm, to the medium 3-4 mm, to the smallest 1-2 mm). Homogenates were slowly aspirated 10 times in each pipette and then were incubated at 37 °C for 10 min under slow and continuous rotation. Samples were then centrifuged (1200 rpm; 10 min at room temperature, RT) in an HBSS solution (1X; #55037 C; Sigma) and the supernatants discarded.

#### Myelin removal

The pellet was resuspended in an enzymatic solution from the kit before being incubated with the magnetic myelin removal beads (#130-096-731; Miltenyi Biotec) for 15 min at 4 °C. Cells were then washed by using buffer myelin removal solution and then centrifuged (1249 rpm; 10 min at RT). The pellet was re-suspended in the same solution and loaded onto the MS column, placed in the magnetic field separator. The eluted solution (unlabeled cells) was collected, containing cells without the myelin, while the magnetically labeled cells (cells with myelin) were retained on the MS column. The eluted solution was centrifuged in order to eliminate dead cells (1249 rpm; 5 min at RT).

#### Anti-ACSA-2 microbeads labeling

The pellet was re-suspended in buffer myelin removal solution with FcR blocking solution. Samples were incubated 10 min at 4 °C before addition of the anti-ACSA-2 (Astrocyte Cell Surface Antigen 2 [39] microbeads (#130-097-679; Miltenyi Biotec) and the solution was re-incubated for 15 min at 4 °C then centrifuged (1200 rpm; 10 min at 4 °C). The pellet was re-suspended in buffer myelin removal solution and loaded onto the MS column, placed in the magnetic field of the MACS^©^ separator. The magnetically labeled ACSA-2 cells were retained within the column, while the unlabeled cells ran through and were eluted. To increase purity, the positively selective fraction was separated over a second MS column.

#### Anti-CD11b microbeads labeling

The previously unlabeled cells eluted were centrifuged (1249 rpm; 5 min at 4 °C) and the pellet re-suspended in buffer myelin removal solution before addition of the anti-CD11b microbeads (#130-097-142; Miltenyi Biotec). Samples were incubated 15 min at 4 °C and then centrifuged (1200 pm; 10 min at 4 °C). The pellet was re-suspended in buffer myelin removal solution and loaded onto the MS column, placed in the magnetic field of the MACS^©^ separator. The magnetically labeled CD11b-positive cells were retained within the column, while the unlabeled cells ran through and were eluted. To increase purity, the positively selective fraction was separated over a second MS MACS^©^ column.

In order to perform the sequencing analysis, RNA was extracted from the ACSA-2 positive and CD11b positive cell suspensions corresponding to astrocytic cells or microglial cells, respectively. RNA sequencing was performed as indicated in the Supplementary methods.

##### 3D glial cell morphology

**Fig. 2:**
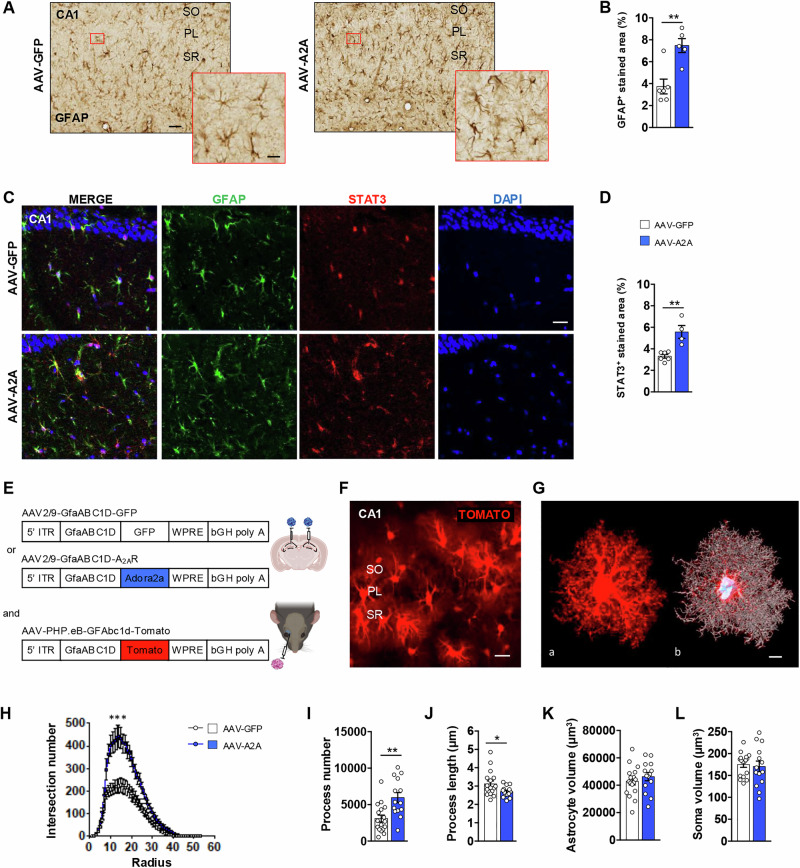
A_2A_R upregulation in hippocampal astrocytes impacts their reactivity and complexity. **A** Representative images of GFAP immunostainings in the CA1 of AAV-GFP (left panel) and AAV-A2A (right panel) animals (scale bar=100 μm; magnification in red inserts scale bar=50 μm). **B** Quantification indicates a significant increase of the GFAP^+^ staining in the hippocampal CA1 astrocytes of AAV-A2A mice as compared to AAV-GFP controls (N = 5-6 mice/group; 3-5 sections analyzed/animal; **P < 0.01 vs. AAV-GFP; Mann-Whitney test). **C** Representative images of GFAP (green), STAT3 (red) and DAPI (blue) co-immunostainings in the hippocampal CA1 area of AAV-GFP (upper panel) and AAV-A2A (lower panel) animals (scale bar=50 μm). **D** A significant increase of the STAT3^+^ staining in the GFAP^+^ astrocytes of AAV-A2A mice was observed as compared to AAV-GFP controls in the hippocampal CA1 area (N = 4-6 mice/group; 2-3 sections analyzed/animal; **P < 0.01 vs. AAV-GFP; Student’s t-test). **E**, **F** A retro-orbital injection of an AAV-PHP.eB carrying tdTomato gene under the control of the GfaABC1D astrocytic promoter in both AAV-GFP and AAV-A2A animals was performed **(E)** in order to express tdTomato protein in sparse CA1 astrocytes (cytosol and arborization; scale bar=60 μm; **F**). **G** Isolated Tomato^+^ astrocytes of the *stratum radiatum* CA1 sublayer were imaged by high-resolution imaging (a) and 3D-reconstructed using Imaris software (b). **H–J** An increase in the intersection number, as shown by Sholl analysis (***P < 0.0001 vs. AAV-GFP, Two-Way ANOVA; **H**) as well as of the number of the astrocytic processes (N = 14-17 astrocytes from N = 5-6 mice/group; **P < 0.01 vs. AAV-GFP, Student’s t-test; **I**) was found in the *stratum radiatum* CA1 astrocytes of AAV-A2A condition as compared to the AAV-GFP controls. That was accompanied by a reduced process length (N = 14-17 astrocytes from N = 5-6 mice/group; *P < 0.05 vs. AAV-GFP; Student’s t-test; **J**) **(K–L)** No change in the overall astrocyte volume (P = 0.40; **K**) or the astrocytic soma volume (P = 0.74; **L**) could be observed AAV-A2A animals as compared to the AAV-GFP controls (N = 14-17 astrocytes from N = 5-6 mice/group). Values are represented as mean ± SEM. *Stratum oriens* (SO); pyramidal layer (PL); *stratum radiatum* (SR) sublayers.

At the time of AAV-A2A and AAV-GFP stereotaxic injections, a dedicated group of mice also received a retro-orbital (RO) venous sinus delivery of an AAV-PHP.eB at 5.10^10^ vg in 50 μl total volume using a U100 insulin syringe (BD micro-fine 0.3 mL, 30-gauge needle). This AAV with a PHP.eB capsid (AAV-PHP.eB-GfaABC1D-Tomato or AAV-PHP.eB), known to cross the blood-brain barrier after intravenous injection [40], was used to express the fluorescent Tomato protein in sparse astrocytes to allow 3D imaging afterwards (Fig. 2E–G). For these mice, brains were removed and post-fixed in 4% PFA (in PBS; pH 7.4) for 24 h at 4 °C and stored into PBS-azide until process. They were cut using a Leica vibratome (parameters: 0.12 speed and amplitude of 2 μm) at a thickness of 100 μm according to the coronal axis. The astrocytic 3D-reconstructions were performed thanks to the confocal microscope and the imaging software Imaris (Bitplane, USA).

#### Astrocytic 3D-reconstruction and analysis

The 100 μm mouse free-floating vibratome sections were directly mounted on slides and coverslips (High performance thickness 1^1/2^ and diameter 0.170 ± 0.005 mm; Zeiss). Tomato-positive astrocytes of the CA1 area were imaged on a Zeiss LSM-710 confocal microscope taking-up to 50 stacks at 0.43 μm steps with a x63 oil objective (optimal frame size of 1348). The complex arborization of the CA1 astrocytes was 3D reconstructed using the “filament tracer” rendering function in Imaris and 3D analyzed using the Imaris plug-in “X-tension” (4-6 astrocytes/mice). Then the 3D cell and soma volumes as well as the number, the length of the processes and the Sholl intersections were analyzed. Sholl intersections are the number of times a cell extension in a 2D representation intersects the concentric circles, or radius, positioned at a 1 μm interval and starting from the cell soma.

#### Microglia 3D-reconstruction and analysis

After performing Iba1 immunofluorescence on 35 μm mouse free-floating sections, Iba1^+^ cells of the CA1 area were imaged using a Zeiss Spinning Disk high-resolution microscope taking-up to 15 z-stacks at 1.5 μm steps with a 40x oil objective. The complex arborization of the CA1 microglia was 3D reconstructed using the “filament tracer” rendering function in Imaris and 3D analyzed using the Imaris plug-in “X-tension” (10-15 microglia/mice). Then the Sholl intersections were analyzed.

##### Preparation of synaptosomes

One hippocampus per mouse was homogenized in 200 μl of Tris buffer (pH 7.4) containing 10% sucrose using a Potter-Elvehjem on ice. Protein concentrations were quantified using the bicinchoninic acid assay (Pierce) and diluted in Tris buffer (pH 7.4) containing 10% sucrose to obtain a final concentration of 4 ug/μl. 200 μl of Syn-PER™ Synaptic Protein Extraction Reagent (#87793; Thermofisher, France) was then added to 100 μl of the protein homogenates. The samples were homogenized again using a Potter-Elvehjem and then centrifuged (1200 g; 10 min at 4 °C). Cell debris were discarded, supernatants centrifuged (15000 g; 20 min at 4 °C) and the resulting pellets resuspended in 100 μl of Syn-PER™ solution. These samples were stored at −20 °C until use. Related Western blot analysis is described in the Supplementary methods.

##### Determination of Neuronal excitability using DREADD

In order to evaluate the neuronal excitability in animals with astrocytic A_2A_R upregulation, a serotype 5 AAV carrying hM3Dq DREADD receptor (Designer Receptor Exclusively Activated by Designer Drugs) and mCherry genes, under the control of the neuronal CaMKII (Ca^2+^/calmodulin-dependent protein kinase) promoter, was used (AAV5-CaMKII-hM3Dq-mCherry or AAV-hM3Dq). The AAV-hM3Dq was bilaterally co-injected with the AAV-A2A or AAV-GFP in the CA1 hippocampus area at the concentration of 1.10^9^ vg/μl with a total volume of 2 μl per injection site at a rate of 0.25 μl/min (N = 5/group). Two months following the AAV stereotaxic injections, mice were i.p. administrated with clozapine-N-oxide (CNO; 5 mg/kg), an hM3Dq exogeneous and synthetic ligand, or saline (NaCl; 0.9%) as a control, 90 min before sacrifice (as described above) to acutely activate the hM3Dq-DREADD receptor and evaluate the immediate early genes (IEGs) expression by mRNA and immunohistochemical analysis. To validate the neuronal expression of hM3Dq-DREADD receptor in CA1, we performed an immunohistochemical amplification of the mCherry protein with an anti-RFP antibody for proper visualization. mCherry and RFP stainings cannot be differentiated as both emit at close wavelength. We used this methodology on purpose allowing to reveal better the localization of hM3Dq tagged with RFP. RNA extractions and RT-qPCR from whole hippocampi were performed as described previously [16]. Sequences of primers used in this study are given in Supplementary Table 1. Cyclophilin A was used as a reference housekeeping gene for normalization. Immunohistological and analysis procedures regarding these samples are described in the Supplementary methods.

### Electrophysiological and analysis procedure

#### Acute hippocampal slices

Hippocampal slices were prepared from six-month-old AAV-GFP and AAV-A2A mice. Animals were anaesthetized with isoflurane, humanely killed by cervical dislocation and decapitated. After dissection of the hippocampi, 350-μm-thick transverse hippocampal slices were cut with a Leica 1200S vibratome in an oxygenated (5% CO_2_ and 95% O_2_) ice-cold protective extracellular solution containing (in mM): 93 NMDG, 20 HEPES, 2.5 KCl, 1.2 NaH_2_PO_4_, 30 NaHCO_3_, 2 thiourea, 25 D-glucose, 5 sodium ascorbate, 3 sodium pyruvate, 0.5 CaCl_2_ and 10 MgCl_2_ (pH 7.3, 310 mOsm). After cutting, slices were transferred to the NMDG-HEPES solution for 5-6 min at 33 °C and then incubated at 33 °C for 30 min in regular artificial cerebrospinal fluid (aCSF) containing (in mM): 2.5 KCl, 126 NaCl, 26 NaHCO_3_, 1.25 NaH2PO_4_, 1 sodium pyruvate, 20 mM D-glucose, 2 CaCl_2_, and 1 MgCl_2_ (pH 7.4, 310 mOsm). Slices were maintained at RT (22–24 °C) for up to 5 h in the regular oxygenated aCSF before performing experiments.

#### Electrophysiological recordings

Slices were transferred to a recording chamber placed on the optic path of an upright microscope (Olympus BX51) and perfused with regular oxygenated (5% CO_2_ and 95% O_2_) aCSF (articificial cerebrospinal fluid) at 3 ml/min at a temperature of 32–33 °C. Signals were acquired with Axopatch 200B amplifiers coupled to DigiData 1322 interface, using Clampex 10.5 (Molecular Device). Local field potentials (LFPs) were recorded with a glass micropipette (1MΩ) filled with aCSF and placed in the stratum radiatum of CA1. Signals were acquired in Current Clamp, low-pass filtered at 1 kHz and digitized at 10 kHz. The excitability of the hippocampal network was tested by inducing neuronal burst activities with aCSF containing 4-AP (4-Aminopyridine; 200 μM; HELLOBIO HB1073) and low Mg2+ (20 μM). Slices were first recorded in control aCSF before switching to the low Mg^2+^/4-AP solution. The burst frequency was averaged over 10-14 min from the first burst.

##### Statistical analysis

Behavioral experiments and ll immunohistochemical analysis were conducted by an experimenter blinded to the experimental group. The results were expressed as the mean ± SEM. Data normality was tested using Shapiro-Wilk test. If normality was confirmed, differences between groups were generally determined using either Student’s t-test, One-Way ANOVA followed by a Tukey’s post-hoc test or by two-way ANOVA. Otherwise, data were tested using a Mann-Whitney test. All statistical analyzes were performed with GraphPad Prism version 10 software. A p-value (P) of less than 0.05 was considered significant.

## Results

### Astrocytic upregulation of A_2A_R in the mouse CA1

To determine the impact of astrocytic A_2A_R upregulation in the mouse hippocampus, we bilaterally injected dedicated adeno-associated viral (AAV) vectors allowing the selective expression of A_2A_R (AAV-A2A) or GFP (AAV-GFP), taken as a control, in the CA1 astrocytes of two-month-old C57Bl6/J mice and animals were studied 2 months post-injection (Fig. 1A). Immunohistochemistry using antibodies against A_2A_R or GFP allowed to visualize their hippocampal levels in the different CA1 sublayers i.e., *stratum oriens* (SO), pyramidal layer (PL) and *stratum radiatum* (SR) (Fig. 1B; Supplementary Figure 1). Co-immunostainings using antibodies directed against astrocytic (GFAP, Sox2, S100β), neuronal (NeuN) and microglial (Iba1) markers showed exclusive expression of A_2A_R by astrocytes, as expected (Fig. 1C).

### Astrocytic impact of A_2A_R upregulation on reactivity and morphological complexity

Astrocytes can adopt a reactive phenotype which is notably characterized by an increased expression of GFAP, an intermediate filament protein. We found a significant increase of the GFAP^+^ staining in the CA1 of AAV-A2A mice as compared to the AAV-GFP control (+ 100.2 ± 38.3%; P = 0.0087; Mann-Whitney test; Fig. 2A, B). Recent studies have identified the JAK2/STAT3 signaling pathway as a central player in the induction of the astrocytic reactive state in pathological conditions such as Alzheimer’s disease (AD) and Huntington’s disease [41]. Accordingly, we performed a GFAP/STAT3 co-immunostaining and observed a significant increase of STAT3^+^ staining within the GFAP^+^ astrocytes of the CA1 area in the AAV-A2A condition as compared to the AAV-GFP control (+ 68.3 ± 35.9%; P = 0.0024; Student’s t-test; Fig. 2C, D). Moreover, astrocytes continually adapt to their environment and exhibit morphological changes eventually associated with their reactive state [42]. In order to assess more finely the morphological changes of astrocytes upon A_2A_R upregulation, a 3D-reconstruction of the latter was performed, following a retro-orbital injection of an AAV-PHP.eB allowing the selective expression of tdTomato in sparse CA1 astrocytes (cytosol and arborization), of both AAV-A2A and AAV-GFP-injected mice (Fig. 2E, F). We thus imaged isolated tdTomato^+^ astrocytes in the CA1 *stratum radiatum* sublayer by high-resolution imaging and performed their 3D-reconstruction using Imaris software (Fig. 2G). Our results indicated a significantly higher number of intersections, as shown by Sholl analysis, consistent with a higher number of astrocytic processes in AAV-A2A vs. AAV-GFP mice (Fig. 2H, I). A decreased astrocytic processes length was also observed (Fig. 2J) without change of the overall astrocyte or soma volumes (Fig. 2K–L). Together, these results support that astrocytic A_2A_R upregulation promotes astrocyte reactivity and complexity.

### Aging-like molecular signature induced by astrocytic A_2A_R upregulation

**Fig. 3:**
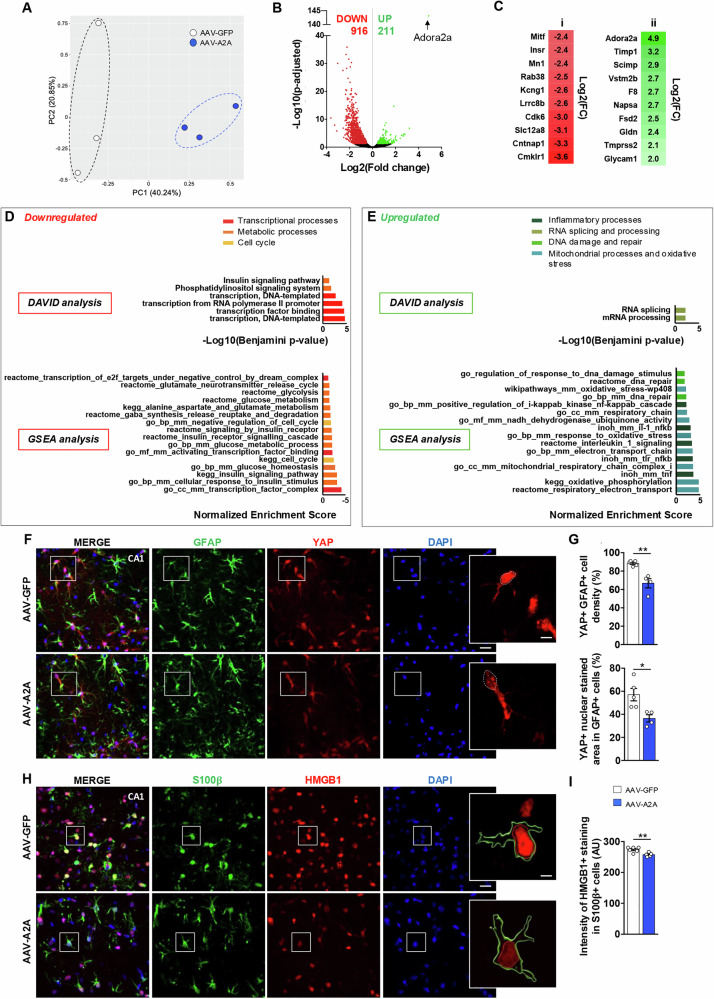
Astrocytic A_2A_R upregulation in hippocampus is associated with an aging-like astrocyte signature. **A** The principal component analysis (PCA) performed on RNA-seq datasets shows that the two conditions AAV-GFP (white dots) and AAV-A2A (blue dots) are nicely separated on the first Principal component (PC1). **B** Volcano plot representing the deregulation of 1127 genes in the astrocytes of AAV-A2A vs. AAV-GFP mice using cut offs of |Log2 fold-change (FC)|>0.32 and adjusted P-value (Padj)<0.05. 916 genes were found downregulated and 211 upregulated, including the *Adora2a* gene. **C** Top 10 genes significantly downregulated (red) (i) and upregulated (green) (ii) in hippocampal astrocytes of AAV-A2A animals vs. AAV-GFP controls with |Log2 fold-change (FC)|>0.32 and Padj<0.05. **D**, **E** Functional enrichment analysis of GO Biological Process annotations using DAVID (upper panels) and GSEA analysis (lower panels) associated to **(D)** downregulated genes and **(E)** upregulated genes. **F** Representative images of GFAP (green), YAP (red) and DAPI (blue) co-immunostainings in the CA1 of AAV-GFP (upper panel) and AAV-A2A (lower panel) groups (scale bar=50 μm). Inserts represent YAP^+^ nuclei (dashed lines) in both groups (scale bar=10 μm**). G** A significant decrease in the percentage of YAP^+^ cells among GFAP^+^ astrocytes as well as a reduction of the YAP staining within the nuclei of GFAP^+^ astrocytes were observed in the CA1 hippocampal area of AAV-A2A mice vs. AAV-GFP controls (N = 4-5 mice/group; 3 sections analyzed/animal; *P < 0.05, **P < 0.01 vs. AAV-GFP, Student’s t-test). **H** Representative images of S100β (green), HMGB1 (red) and DAPI (blue) co-immunostainings in the CA1 of AAV-GFP (upper panel) and AAV-A2A (lower panel) groups (scale bar=50 μm). Inserts represent HMGB1 staining (red lines) in S100β^+^ cells (green lines) in both groups (scale bar=7 μm). **I** A significant decrease of the HMGB1 staining intensity in S100β^+^ astrocytes was observed in the CA1 hippocampal area of AAV-A2A mice vs. AAV-GFP controls (N = 4-5 mice/group; 3-4 sections analyzed/animal; **P < 0.01 vs. AAV-GFP, Student’s t-test).

To gain molecular insights on the impact of astrocytic A_2A_R upregulation, we performed a RNA sequencing analysis of hippocampal astrocytes of AAV-A2A and AAV-GFP-injected mice. To do so, we used magnetic cell sorting with ACSA-2 (Astrocyte Cell Surface Antigen 2 [39]; Supplementary Figure 2A) magnetic microbeads in order to isolate astrocytes from freshly dissected hippocampi. Following RNA sequencing, and using an available database [43], we first validated our enrichment procedure by comparison of the expression of known genes specifically expressed by astrocytes with genes expressed by other cell types (Supplementary Figure 2B). The first Principal Component (PC1), which explained 40.24% of the variance, nicely separated our experimental conditions (Fig. 3A). We found 1127 differentially expressed genes (DEG) in the AAV-A2A condition as compared to the AAV-GFP control, 916 being downregulated and 211 upregulated (|Log2 fold-change (FC)|>0.32; Adjusted P-value (Padj)<0.05; Fig. 3B; list of DEG available on Supplementary Table 3). The top ten of downregulated genes is given on Fig. 3Ci. Functional enrichment analysis provided by DAVID and GSEA analysis indicated that downregulated astrocytic genes in AAV-A2A mice are associated with several metabolic processes such as insulin signaling as well as glucose and glutamate metabolism (Fig. 3D; Supplementary Figure 3). The GSEA analysis particularly indicated a downregulated pathway noted as “glutamate_neurotransmitter_release_cycle”, containing several downregulated genes (Slc1a1, Slc1a2, Slc1a3, Slc1a6, Slc1a7) involved in the regulation of glutamate reuptake by astrocytes, among which Slc1a2, encoding GLT1 transporter. Further, our astrocytic RNA-Seq data also indicate that A_2A_R upregulation leads to a significant reduction of hippocampal mRNA levels of glutamine synthetase (GS), involved in the recycling of glutamate into glutamine (Log2 FC = -1.75; Padj=2.37^E-11^). Downregulation of both GLT1 and GS have been validated by western blots (Supplementary Figure 4A, B). We also observed that astrocytic A_2A_R upregulation exerts a significant impact on genes governing/regulating transcriptional processes (Fig. 3D; Supplementary Figure 3). Regarding upregulated genes, as expected, we found A_2A_R (*Adora2a*). We also observed the upregulation of *Timp1* (Log2 FC = 3.19; Padj=1.59^E-07^), a gene encoding a metalloproteinase inhibitor, being involved in astrocyte reactivity as well as in neurodegenerative conditions [44, 45] (Fig. 3Cii). GSEA analysis indicated that upregulated genes were associated with inflammatory processes (NFκB, TNFɑ and Il1) as well as oxidative stress and DNA repair pathways (Fig. 3E; Supplementary Figure 3), previously linked to cellular processes associated with aging [46, 47]. One of the hallmarks of aging, cellular senescence, has been particularly linked with astrocytes in AD [48, 49]. Interestingly, it has been recently demonstrated that astrocytic senescence associates with impairment in the activity of YAP (Yes1 Associated Transcriptional Regulation), being required for the expression of CDK6 (Cyclin-Dependent Kinase 6) [50]. Accordingly, our data highlighted significant reductions in the mRNA levels of *cdk6* (Log2 FC = −3.03; Padj=8.85^E-31^; Figure 3Ci) and *yap1*, coding YAP (Log2 FC = −1.12; Padj=6.93^E-05^). In order to validate the impairment of YAP, instrumental to astrocyte senescence [50], we performed co-immunostainings against YAP and GFAP (Fig. 3F). Our data showed a significant reduction in the percentage of YAP^+^ cells among GFAP^+^ cells in AAV-A2A vs. AAV-GFP mice (-25.0 ± 11.2%; P = 0.002; Student’s t-test) as well as a reduction of the nuclear YAP staining in GFAP^+^ astrocytes (-45.0 ± 11.5%; P = 0.0183; Student’s t-test) in the CA1 of AAV-A2A vs. AAV-GFP mice (Fig. 3G). We also found that the volume of astrocyte nuclei was significantly enlarged in YAP^+^ GFAP^+^ of A_2A_R expressing astrocytes as compared to GFP controls ( + 22.8 ± 8.0%; P = 0.0344; Student’s t-test; 6-8 nuclei per animals; N = 4-5/group; not shown), another hallmark of senescence [51, 52]. To further validate the aging-like/senescence phenotype in hippocampal astrocytes following A_2A_R upregulation, we also assessed HMGB1, a marker commonly used in the literature and lost in senescence [52], belonging to the High Mobility Group Box1 proteins family and involved in the regulation of chromatin structure [53]. Our data demonstrate a significant reduction of HMGB1 staining in the S100β^+^ astrocytes upon A_2A_R astrocytic upregulation as compared to GFP controls (Fig. 3H, I). Taken together, these data suggest that A_2A_R upregulation in hippocampal astrocytes is sufficient to induce a phenotype related to aging and senescence. We therefore compared our transcriptomic data with the signature of astrocytes isolated from aged mice and also to a list of “astrocytic senescence-related genes” extracted from the literature (Supplementary Figure 5A; Supplementary Table 4; Supplementary Table 5). We identified an overlap of 33 genes (out of 358) that showed similar variation in the AAV-A2A animals when compared to the “aged” signature, and 13 genes (out of 62) from the “astrocytic senescence-related genes” signature (Supplementary Figure 5A).

### Astrocytic upregulation of A_2A_R increases neuronal excitability

**Fig. 4:**
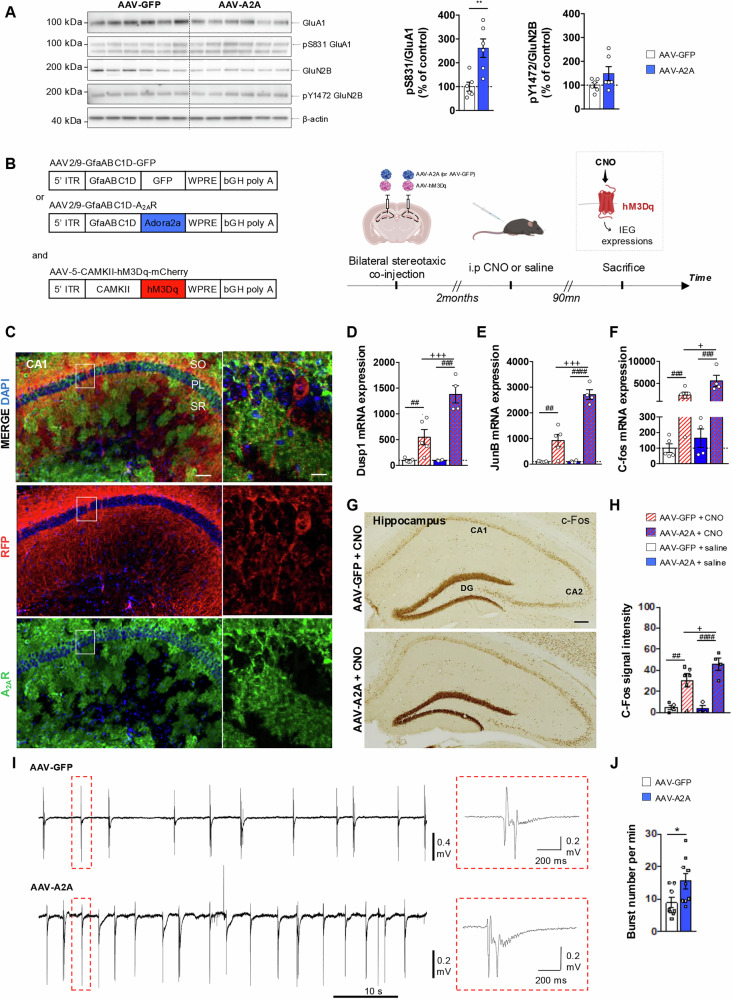
Astrocytic A_2A_R upregulation in hippocampus alters neuronal activation. **A** Phosphorylation of the GluN2B subunit (NMDAR) at Y1472 and of the GluA1 subunit (AMPAR) at pS831 in synaptosomal fraction of AAV-GFP and AAV-A2A hippocampi. Quantification shows a significant increase of pS831 GluA1 in AAV-A2A vs. AAV-GFP mice (*P < 0.05; **P < 0.01 vs. AAV-GFP; Student’s t-test; N = 6/group). **B** Bilateral injection of an AAV5 carrying the hM3Dq gene under the control of the CAMKII neuronal promoter allows the activatory Gq-coupled DREADD-mCherry receptor expression in hippocampal neurons of AAV-GFP and AAV-A2A animals. Two months following the AAV co-injections, an i.p administration of the exogeneous and synthetic hM3Dq ligand (clozapine-N-oxyde, CNO; 5 mg/kg), or saline (NaCl; 0.9%) as a control, induced neuronal activation measured by the immediate early genes (IEGs) expression. **C** Representative images of A_2A_R (green), RFP-amplified hM3Dq-mCherry (red) and DAPI (blue) co-immunostainings in CA1 hippocampal area (scale bar=200 μm; magnification: scale bar=10 μm). **D–F** The mRNA expression of *Dusp1* **(D),** *JunB* **(E)** and *c-fos* **(F)** was found significantly higher in CNO-treated vs. saline-treated animals. Notably, IEGs expressions were significantly enhanced in AAV-A2A as compared to AAV-GFP mice in CNO-treated conditions. (N = 4-5 mice/group; ^##^P < 0.01; ^###^P < 0.001: CNO-treated vs. respective saline-treated animals; ^+^P < 0.05; ^+++^P < 0.001 in AAV-A2A + CNO-treated animals vs. AAV-GFP ^+^ CNO-treated; One-Way ANOVA followed by Tukey’s post-hoc test). **G** Representative images of c-Fos immunostaining in the CA1, CA2 and DG regions of hippocampus in AAV-GFP (upper panel) and AAV-A2A (lower panel) animals treated with CNO (scale bar=400 μm). **H** As expected, the intensity of c-Fos was significantly higher in CNO-treated vs. saline-treated animals. c-Fos immunostaining intensity was found significantly higher in AAV-A2A + CNO as compared to AAV-GFP + CNO controls. (N = 4-5 mice/group; 2 sections analyzed/animal; ^##^P < 0.01; ^###^P < 0.001: CNO-treated vs. respective saline-treated animals; ^+^P < 0.05 in AAV-A2A + CNO-treated animals vs. AAV-GFP + CNO-treated; One-Way ANOVA followed by Tukey’s post-hoc test). **I** Recordings of local field potentials showing the bursting activity of the CA1 region of hippocampal slices of AAV-GFP (upper trace) and AAV-A2A (lower trace) mice during application of 4-AP/Low Mg^2+^ -containing extracellular solution. Insets (red dotted rectangles) show a magnification of a single burst in both groups. **J** The burst frequency was significantly higher in AAV-A2A animals as compared to the AAV-GFP controls (N = 8-9 slices/group from 3-4 mice/group; *P < 0.05 vs. AAV-GFP using Mann-Whitney test). Values are represented as mean ± SEM.

Astrocytes regulate glutamate neurotransmission and synaptic plasticity. Interestingly, the above-mentioned transcriptomic data supported that astrocytic upregulation of A_2A_R significantly impacts genes involved in glutamate release, in line with previous data reporting that A_2A_R tightly controls (inhibits) glutamate reuptake by astrocytes [5, 6]. This led us to address the effect of A_2A_R upregulation on neuro-astroglial communication and particularly markers of neuronal excitability. We first prepared hippocampal synaptosomes from AAV-GFP and AAV-A2A mice to evaluate the phosphorylation of glutamatergic receptor subunits important for the synaptic trafficking and conductance of NMDA and AMPA receptors, focusing on the phosphorylation of NR2B at Y1472 and of S831 of GluA1 [54, 55]. While pY1472 GluN2B/GluN2B ratio did not significantly change in AAV-A2A mice, we observed a significant increase ( + 161.4 ± 94.7%; P = 0.003; Student’s t-test) of the synaptosomal pS831 GluA1/GluA1 ratio as compared to AAV-GFP animals (Fig. 4A). Then, we used a DREADD chemogenetic tool to determine whether A_2A_R upregulation in astrocytes was prone to affect neuronal activation. We jointly expressed the activatory Gq-coupled DREADD receptor (hM3Dq; with an mCherry reporter) in CA1 neurons with A_2A_R or GFP in CA1 astrocytes (Fig. 4B). A co-immunostaining against A_2A_R and RFP, in order to amplify and visualize the hM3Dq-mCherry signal, confirmed the astrocytic expression of A_2A_R (green; Fig. 4C) and the neuronal expression of the hM3Dq (red; Fig. 4C). Two months post-AAV injections, we intraperitoneally injected CNO, the exogeneous and synthetic ligand of hM3Dq, or saline as control, to selectively activate hippocampal neurons. Ninety minutes later, we evaluated mRNA levels of immediate early genes (IEGs) as an indicator of neuronal activation by CNO injection (Fig. 4B). As expected, our data demonstrated a significant hippocampal increase of *Dusp1*, *JunB* and *c-fos* levels in CNO-treated (vs. saline) animals (Fig. 4D–F). Notably, the magnitude of IEGs activation was significantly larger in AAV-A2A mice as compared to AAV-GFP animals in CNO-treated condition (Fig. 4D–F). C-Fos immunohistochemistry confirmed qPCR data, as shown in Fig. 4G, H (controls are shown in supplementary Figure 6). In addition, we compared the excitability of acute hippocampal slices from of AAV-GFP and AAV-A2A mice by recording the neuronal bursting activity induced by a 4-AP/Low Mg^2+^-containing extracellular solution. By blocking potassium channels (4-Aminopyridine, 4-AP) as well as promoting the activation of calcium channels and NMDA receptors (low Mg^2+^), this solution favors neuronal excitability and synaptic activity leading to the appearance of spontaneous neuronal bursts. Local field potential recordings in CA1 showed that the mean frequency of hippocampal bursts was significantly higher in AAV-A2A mice than in AAV-GFP mice (Fig. 4I, J; AAV-GFP: 8.78 ± 1.6 bursts/min vs. AAV-A2A: 15.56 ± 2.38 bursts/min; AAV-GFP: 8.78 + /-1.6 bursts/min; P = 0.036, Mann-Whitney test).

### Astrocytic A_2A_R upregulation alters microglial phenotype

**Fig. 5:**
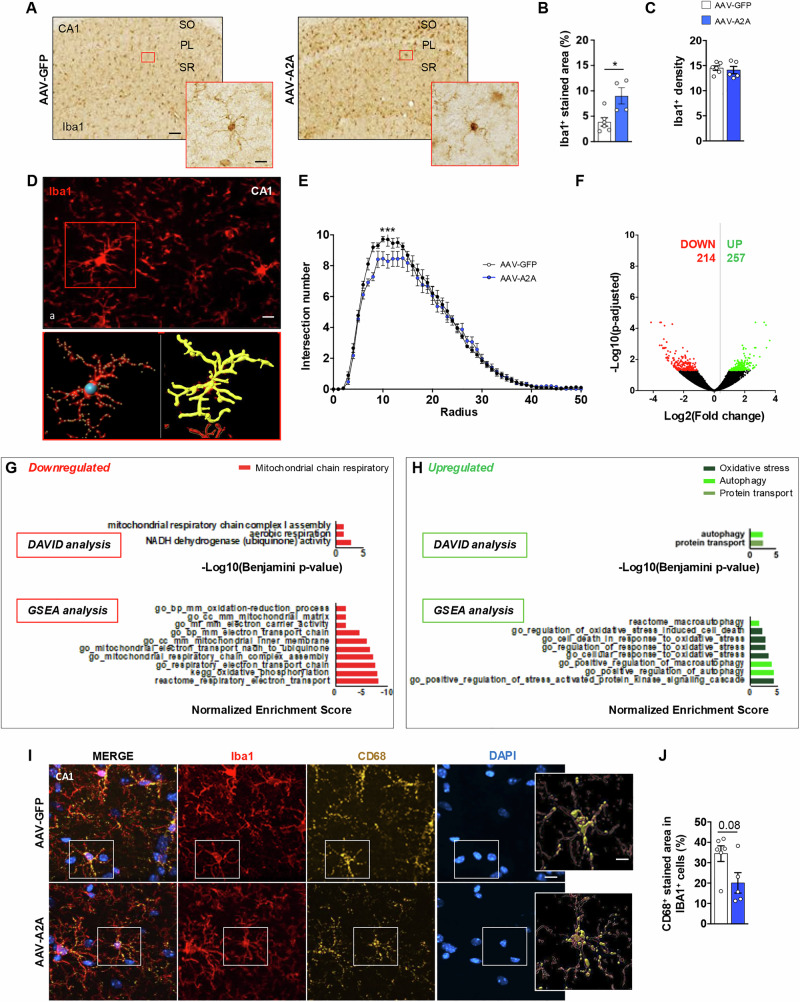
Astrocytic A_2A_R upregulation in hippocampus alters microglial phenotype. **A** Representative images of Iba1 immunostaining in AAV-GFP (left panel) and AAV-A2A (right panel) conditions in the CA1 hippocampal area (scale bar=100 μm; magnifications: scale bar=10 μm). **B**, **C** A significant increase of the Iba1^+^ staining was observed in the hippocampal astrocytes of AAV-A2A condition as compared to the AAV-GFP control (N = 4-6 mice/group; 3-5 sections analyzed/animal; *P < 0.05 vs. AAV-GFP; Student’s t-test; B) with no change in the density of Iba1^+^ cells **(C)**. **D** Representative images of Iba1 immunostaining (a) and 3D reconstruction (b,c) (scale bar=15 μm; magnification: scale bar=2 μm). **E** Decrease in the intersection number (complexity), as shown by Sholl analysis, was found in the microglia of AAV-A2A mice as compared to AAV-GFP controls (N = 10-15 microglia from N = 5 mice/group; Two-Way ANOVA). **F** Volcano plot representing the deregulation of 471 genes (DEG) in the microglia of AAV-A2A vs. AAV-GFP mice using cut offs of |Log2 fold-change (FC) | > 0.32 and adjusted P-value (Padj)<0.05. 214 genes were found downregulated and 257 upregulated. **G**, **H** Functional enrichment analysis of GO Biological Process annotations using DAVID (upper panels) and GSEA analysis (lower panels) associated to **(G)** downregulated genes and **(H)** upregulated genes. **I** Representative images of Iba1 (red), CD68 (yellow) and DAPI (blue) co-immunostainings in the CA1 hippocampal area in AAV-GFP (higher panel) and AAV-A2A (lower panel) groups (scale bar=10μm; magnification: scale bar=2 μm). **J** A trend for a significant reduction of the CD68^+^ staining in the Iba1^+^ cells of the stratum radiatum CA1 sublayer was found in AAV-A2A as compared with AAV-GFP controls (N = 5-6 mice/group; 3-4 sections analyzed/animal; P = 0.08 vs. AAV-GFP; Mann-Whitney test). Values are represented as mean ± SEM.

Besides the link with neurons and considering that A_2A_R upregulation activates a pro-inflammatory signature in astrocytes, we further aimed at determining to which extent such astrocyte changes may impact microglial cells. We first performed immunohistochemistry targeting Iba1, a calcium-binding protein whose expression is upregulated upon microglial activation. (Fig. 5A). Our results showed a significant increase of the Iba1^+^ staining in AAV-A2A animals ( + 131.8 ± 40.9%; P = 0.013; Student’s t-test; Fig. 5B) without change in the density of Iba1^+^ cells (Fig. 5C). Like astrocytes, microglial phenotype exhibits a significant heterogeneity and complexity depending on cellular environment [56]. To capture potential changes of microglial morphology, we performed a 3D-analysis from Iba1 immunofluorescence staining. The Sholl analysis revealed a slight but significant reduction of the microglial complexity in AAV-A2A animals as compared to AAV-GFP controls (P < 0.001; Two-Way ANOVA; Fig. 5D, E). To comprehensively assess the impact of astrocytic A_2A_R upregulation on microglial cells, we conducted RNA sequencing analysis on hippocampal microglia isolated from AAV-A2A- and AAV-GFP-injected mice, using magnetic-activated cell sorting (MACS) with anti-CD11b magnetic microbeads. We first validated our enrichment procedure by comparing the expression of known genes specifically expressed by microglia with genes expressed by other cell types (Supplementary Figure 7A). We found 471 differentially expressed genes (DEG) in the AAV-A2A condition as compared to the AAV-GFP control, 214 being downregulated and 257 upregulated (|Log2 fold-change (FC)|>0.32; Adjusted P-value (Padj)<0.05; Fig. 5F; list of DEGs available on Supplementary Table 6). To determine whether the observed microglial molecular signature was associated with previously characterized neuroinflammatory states, we compared it to established signatures of Disease-Associated Microglia (DAM) and Interferon-Responding Microglia (IRM), but observed no significant overlap (Supplementary Figure 7B). Further, analysis of several homeostatic markers from our RNA-Seq analysis did not highlight any particular changes (Supplementary Figure 7C). Interestingly, functional enrichment analysis provided by DAVID and GSEA analysis indicated a significant decrease in pathways associated with mitochondrial respiratory chain activity, accompanied by an increased autophagic and oxidative stress-related pathways in AAV-A2A mice (Fig. 5G, H). Finally, given that one of the key functions of microglia is to maintain homeostasis -particularly through precise phagocytic activity involved in the clearance of pathogens and misfolded proteins- we assessed the expression levels of CD68, a lysosomal marker associated with phagocytic function. To do so, co-immunostaining against both Iba1 and CD68 was performed and, after 3D analysis, we observed a non-significant decrease of the CD68^+^ staining in the Iba1^+^ cells, suggestive of a reduced microglia phagocytosis in the AAV-A2A animals as compared to the AAV-GFP control (-41.6 ± 32.4%; P = 0.08; Mann-Whitney test; Fig. 5I, J). Together, these data suggest that the upregulation of A_2A_R in astrocytes is sufficient to significantly affect microglial phenotype associated with functional and morphological changes, although this phenotype does not correspond to canonical pathological neuroinflammatory signatures.

### Astrocytic A_2A_R upregulation in CA1 impairs short-term spatial memory and spatial learning

**Fig. 6:**
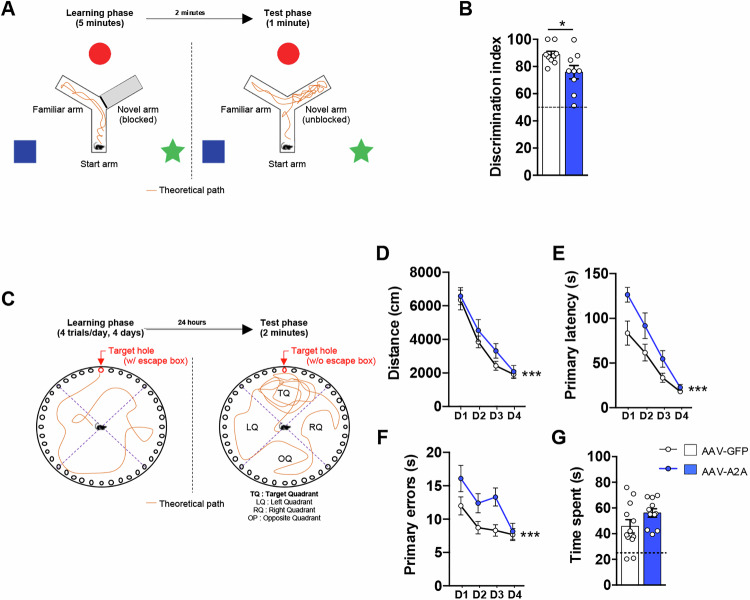
Astrocytic A_2A_R upregulation in hippocampus impairs short-term spatial memory and spatial learning. **A** Schematic representation of the Y-Maze experimental paradigm described in the Material and Methods section. **B** The discrimination index, taken as a measure of the short-term spatial preference for the novel arm in the Y-maze task, was significantly lower for the AAV-A2A animals as compared with AAV-GFP controls (N = 9 mice/group; *P < 0.05 vs. AAV-GFP; Student’s t-test). **C** Schematic representation of the Barnes Maze task experimental paradigm described in the Material and Methods section. **D–F** During the learning phase of the Barnes task, the distance **(D)**, the primary latency **(E)** and the number of primary errors **(F)** were found significantly higher for the AAV-A2A animals as compared to AAV-GFP controls (N = 11-12 mice/group; ***P < 0.001; Two-Way ANOVA). **G** No change regarding the time spent in the target quadrant during the retention phase was found between AAV-A2A and AAV-GFP animals (N = 11-12 mice/group; P > 0.05; Student’s t-test). Values are represented as mean ± SEM.

Finally, to determine whether primary (astrocytic) and secondary (neuronal and microglial) modifications induced by the astrocytic upregulation of A_2A_R in the hippocampus were sufficient to alter memory, we performed two hippocampus-dependent spatial memory tests following an Elevated-Plus Maze evaluation. Elevated-Plus Maze revealed no difference in either the locomotor activity or anxiety-like behavior (percentage of the time spent in the open arms) in animals overexpressing astrocytic A_2A_R (Supplementary Figures 8A–C). Then, short-term spatial memory performance was assessed using the Y-maze test (Fig. 6A). We first observed no difference regarding the distance moved and the velocity (Supplementary Figures 8D, E). A discrimination index, corresponding to the animal preference for the new arm versus the familiar arm during the retention phase, was calculated and showed a preference >50% i.e., vs. chance for both groups (P < 0.0001; One-Sample Student’s t-test), indicating that AAV-GFP and AAV-A2A mice both had a significant preference for the novel arm (Fig. 6B). However, the discrimination index was significantly lower in AAV-A2A animals as compared to AAV-GFP controls, indicating reduced short-term spatial memory (P = 0.03; Fig. 6B). We also evaluated long-term spatial memory performance using the Barnes maze task (Fig. 6C). During the 4 days of the learning, the AAV-A2A group exhibited significantly higher distance (P < 0.001; Two-Way ANOVA), primary latency (P < 0.001; Two-Way ANOVA) and primary errors (P < 0.001; Two-Way ANOVA) to find the goal box as compared to AAV-GFP animals, suggesting altered spatial learning abilities (Fig. 6D–F). During the retention stage, 24 h after the learning, the animals did not show any difference regarding the distance moved or the velocity (Supplementary Figures 8F, G). The percentage of time spent in the target quadrant was significantly higher than 25% i.e., than chance (P = 0.021 in AAV-GFP animals; P < 0.0001 in AAV-A2A animals; Student’s t-test) and similar for both groups, indicating intact spatial memory for both groups (Fig. 6G). Overall, these data highlighted that the multicellular alterations induced by the hippocampal upregulation of A_2A_R in astrocytes ultimately lead to impairments of the short-term spatial memory and learning abilities in mice.

## Discussion

Neuronal and astroglial A_2A_R activation support hippocampal function and memory encoding [1, 2, 9, 18, 32]. A_2A_R expression is upregulated in hippocampal neurons and astrocytes under various allostatic and pathological conditions. Neuronal A_2A_R upsurge during development controls synapse stability [57] whereas in aging it contributes to impaired plasticity and memory decline [17, 58]. Neuronal A_2A_R also rises in several detrimental situations such as chronic stress [19] as well as in Alzheimer’s disease (AD) and other tauopathies [16, 17]. Our recent works in AD models have underscored the instrumental role of neuronal A_2A_R dysregulation in synaptic loss, driven by disrupted neuro-microglia communication [16, 27]. In sharp contrast, although astrocytic dysregulation of A_2A_R has been observed in AD and epilepsy [18, 22], the outcomes and their underlying mechanisms remains largely ill-defined.

The mechanisms underlying A_2A_R upregulation in both neurons and astrocytes are far from understood, possibly multiple and remain to be fully uncovered. Several hypotheses co-exist. The enhancement of synaptic adenosine levels mediated by the CD73 ectonucleotidase could be a trigger for A_2A_R upregulation [59]. Several other molecular pathways could be also at play, A_2A_R expression having been shown dependent on DNA methylation, miRNA as well as the activity of several transcription factors such as CREB, ZBP-89 and Yin Yang-1 (YY1) [60–62]. Therefore, the molecular mechanisms leading to A_2A_R upregulation in pathological conditions have yet to be discovered.

The present study investigates, for the first time, the cellular and molecular in vivo mechanisms triggered by a selective A_2A_R upregulation in hippocampal astrocytes in wild-type mice. Our data demonstrate that the sole upregulation of astrocytic A_2A_R is sufficient to induce multicellular alterations involving primarily astrocytes but also secondarily neuronal and microglial changes, ultimately interfering with learning and memory processes.

### Astrocytic A_2A_R upregulation promotes neuronal hyperexcitability

In response to A_2A_R upregulation, we found that hippocampal astrocytes adopt a so-called reactive phenotype [42, 63], characterized by an increased expression of GFAP, STAT3 and *Timp1*, an inhibitor of metalloproteinase, all being particularly identified as markers common to several neurodegenerative conditions, including AD [41, 44, 64]. Morphologically, this reactivity translates into a greater number of astrocytic processes, and therefore complexity of astrocytic arborization, that may lead to functional modifications, particularly at the tripartite synapses where astrocytes finely regulate neurotransmitter dynamics and neuronal signaling [65, 66]. Indeed, our RNA-Seq and western blot data highlight a reduction of the hippocampal levels of GLT1 and glutamine synthetase (GS) following astrocytic A_2A_R upregulation. These observations are in line with previous data showing an antagonistic interaction between activities of A_2A_R and Na^+^/K^+^ATPase-α2 controlling GLT1-dependent glutamate uptake by astrocytes [6]. Since extracellular glutamate is taken up by astrocytes via GLT1 and subsequently converted to glutamine by GS in the cytoplasm, reduced expression of either may likely impair the glutamate clearance and recycling, thereby promoting neuronal hyperexcitability. While the direct impact of astrocytic A_2A_R upregulation on synaptic glutamate levels needs to be firmly demonstrated, GLT1 and GS changes likely explain: *i)* the higher response of IEGs to chemogenetic neuronal activation in animals exhibiting A_2A_R astrocytic upregulation and *ii)* the higher burst response of hippocampal CA1 neurons to 4-AP/low-Mg^2+^ in slices of A2A animals vs. GFP controls. Such enhanced neuronal excitability is associated to the increased hippocampal levels of phosphorylation at serine 831 of the GluA1 subunit of AMPA receptors, which has been shown to potentiate AMPA receptor ion channel function and to be associated with neuronal hyperexcitability, notably in epilepsy [67, 68]. Our observations could therefore explain the neuronal hyperexcitability associated with A_2A_R upregulation and reported in patients with mesial temporal lobe epilepsy, and also observed in the kaïnate mouse model [22, 59]. They also offer a molecular rationale to explain why the risk of seizures is at least 3 times higher in AD patients (that display astrocytic A_2A_R upreguation [18]) than in healthy controls [69].

### Astrocytic A_2A_R upregulation and the link to metabolism

Changes of hippocampal excitability occur concomitantly with other changes unveiled by our transcriptomic data. In particular, astrocytic A_2A_R upregulation favors miscommunication between astrocytes and neurons by presumably impairing insulin-mediated glucose uptake and astrocytic glycolysis [35, 70]. Notably, in order to evaluate a potential impact of the astrocytic A_2A_R upregulation on the expression of different purinergic-related genes in astrocytes, we checked their expression including P1 and P2 purinergic receptors, ectonucleotidases and nucleoside transporters (Supplementary Table 7). Most of the genes remained unaltered upon astrocytic A_2A_R upregulation. Interestingly, we found a significant increase of Adora2b mRNA levels coding the adenosine A_2B_ receptor (A_2B_R). A recent publication [71] highlighted that stimulation of these receptors activates astrocytic glucose metabolism and the release of lactate, while its astrocytic deletion impairs synaptic function and memory. These data suggest that the astrocytic upregulation of A_2B_R upon A_2A_R astrocytic upregulation might reflect a compensatory mechanism, particularly fitting with the ability of astrocytic A_2A_R upregulation to down-regulate glycolytic-related pathways in astrocytes (per our transcriptomic data) and to promote memory impairments.

### Astrocytic A_2A_R upregulation and the link to aging and AD

Since hippocampal upregulation of A_2A_R has been previously correlated to the pathological development in AD brains and associated cognitive deficits [18], we also attempted to determine whether the specific upregulation of astrocytic A_2A_R could directly promote the establishment of an AD-like astrocytic molecular phenotype. To this end, we compared the astrocytic A_2A_R-related transcriptomic signature identified in our study with those derived from tauopathy (P301S) and amyloidogenesis mouse models (APP/PS1; 5xFAD) [72, 73]. We found a very limited overlap of DEGs (2/54 with P301S; 65/2760 with APP/PS1; 26/526 with 5xFAD; data not shown). Overall, our comparison support that the astrocytic upregulation of A_2A_R in the hippocampus of C57Bl6/J mice is not sufficient to promote a neurodegenerative-like signature. However, among the 43 downregulated genes common to A2A mice and the APP/PS1 model [72], we found common senescence markers such as Glul, coding for GS, as well as Yap1. These observations are in agreement with previous works supporting the establishment of cellular senescence in models of amyloidogenesis [74–76].

More generally, the astrocytic transcriptomic signature of the A_2A_R overexpressing astrocytes unveiled numerous processes associated with an aging-like phenotype characterized by oxidative stress, induction of the NF-κB pathway, DNA repair mechanisms as well as a significant reduction in transcriptional processes and cell cycle. Recent data demonstrated that hippocampal astrocytic senescence associates with a reduction of cdk6 induced by a defective YAP activity, a transcriptional coregulator, in these glial cells. This study also reported that the conditional deletion of YAP in astrocytes was sufficient to promote several hallmarks of astrocyte senescence, including hypertrophic morphology, increased β-galactosidase activity, and upregulation of several senescence-associated genes such as p16, p53 and NF-κB as well as downregulation of Lamin B1 [50]. Interestingly, our data demonstrate a loss of YAP expression in GFAP^+^ astrocytes, as well as a significant reduction of the YAP levels in astrocytic nuclei upon upregulation of A_2A_R. This fits well with the observation that activation of Gs-Protein Coupled Receptors, like A_2A_R, can promote phosphorylation of YAP, preventing its translocation to the nucleus and its action as a transcriptional co-regulator [77]. Further, we identified a significant increase of nuclei size in astrocytes presenting with reduced YAP immunoreactivity, also characteristic of astrocytic senescence [52]. Besides YAP, our data also demonstrate a significant reduction of the HMGB1 staining in astrocytes upon A_2A_R astrocytic upregulation as compared to GFP controls. These data are in agreement with the literature demonstrating a loss of expression of this marker in the astrocytic nucleus before being released during the development of a SASP (senescence-associated secretory phenotype) [49, 78]. The significant reduction, amongst the most downregulated genes, of both GS and cdk6, previously described to be markers of astrocytic senescence [50, 79, 80] is also in agreement with the idea that astrocytic A_2A_R upregulation favors the emergence of a senescent-related phenotype in these glial cells. In agreement, we found some overlap between our transcriptomic dataset and the signatures of astrocytes isolated from aged mice [81] as well as an “astrocytic senescence-related genes” signature extracted from the literature [46, 52, 82]. Overall, our data therefore support that A_2A_R upregulation by hippocampal astrocytes implements several processes associated with astrocyte senescence and aging.

### Astrocytic A_2A_R upregulation and the link to microglial dysfunction

Astrocytic senescence might be linked with the release of molecules associated to SASP, some of which being unveiled in our transcriptomic dataset such as interleukins (i.e. IL-1, IL-33), metalloproteases and metalloproteases inhibitors (i.e. MMP15, Timp1) [83] but also HMGB1. These factors enable paracrine signaling to other astrocytes, but also presumably to surrounding cells such as neurons and microglia. Whether senescent A_2A_R astrocytes favor the senescence of surrounding neurons and microglia has not been specifically addressed in our study and further investigations are warranted. However, our data particularly show that the sole astrocytic upregulation of A_2A_R is sufficient to promote microglial reactivity and morphological changes, i.e., a reduction in the number of processes and therefore of microglial complexity as well as a reduction of CD68, a marker of microglial lysosomal activity and phagocytosis. These morphological and functional microglial changes have been associated with hippocampal aging and neurodegeneration [84]. Our observations, showing a causal link between astrocytic A_2A_R and microglia are also in line with data previously obtained in a mouse model of Sandhoff disease, showing an association between astrocytic upregulation of A_2A_R and microglial activation [34] Interestingly, the transcriptomic signature of microglial cells from mice with astrocytic upregulation of A_2A_R supports the idea that microglia did not adopt a pro-inflammatory/disease-related phenotype and did not lose their homeostatic phenotype. Dysregulated microglial pathways following astrocytic A_2A_R upregulation rather suggested a significant decrease in mitochondrial respiratory chain activity, accompanied by increased autophagy and oxidative stress. In fact, these molecular changes closely resemble those previously described in hypoxia and stroke. Previous works highlighted that in these particular conditions, microglia adopt a reduction in their phagocytic activity associated with the activation of autophagy-related processes [85]. We cannot also exclude the possibility that the presumed release of HMGB1 by astrocytes as part of the SASP may directly contribute to the microglial morphological and molecular changes as supported by several studies [86–89]. Further, several hallmarks of brain aging have been suggested to render neuronal circuits vulnerable to hyperexcitability [90], in line with our DREADD and electrophysiological data. Interestingly, besides a direct consequence of astrocytic changes on neurons, microglial impairments notably the loss of phagocytic abilities, suggested by the reduction of CD68, have been also described in models of neuronal hyperexcitability [91, 92], fitting again with our DREADD and electrophysiological data. Therefore, the triggering of neuronal senescence by astrocytic A_2A_R upregulation as previously described by our team remains a possibility [93, 94].

## Conclusion

Taken together, our data indicate that the upregulation of A_2A_R in hippocampal astrocytes promotes multicellular impairments that impact the hippocampal neuronal network functionality, the astrocyte-neuron coupling and microglial function all previously identified as mandatory for the regulation of memory processes [70, 95, 96]. In accordance, we demonstrate that the upregulation of astrocytic A_2A_R in the hippocampus is sufficient to promote spatial learning and memory alterations. This observation is in line with a previous work from Orr et al. [18] showing that the conditional removal of A_2A_R in astrocytes improved memory in aged mice while the chemogenetic activation of astrocytic Gs-coupled signaling impaired it. However, these data seem controversial with other reports that rather support the detrimental impact of astrocytic A_2A_R deletion in memory settings [32, 97]. Therefore, a tight control of astrocytic A_2A_R might be necessary to ensure proper cognitive functions, especially in pathological conditions. Considering the clinical interest of A_2A_R targeting drugs in AD and epilepsy, this warrants further mechanistic studies.

## Supplementary information

Supplementary methods

Supplmentary figures

Supplementary data - uncropped gels

Supplementary_Tables_3_4_5_6_7

Supplementary_Table_1

Supplementary_Table_2

## Data Availability

Data supporting our study findings are available from the corresponding author upon reasonable request.
