# Supplementary material for: Upregulation of adenosine A2A receptor in astrocytes is sufficient to trigger hippocampal multicellular dysfunctions and memory deficits
Source: Mol Psychiatry. 2025 Jul 23;30(11):5300–14. doi: 10.1038/s41380-025-03115-9 (PMC12532706; doi:10.1038/s41380-025-03115-9)
Supplement: Supplementary file 1 — Supplementary methods [file 41380_2025_3115_MOESM1_ESM.docx]

**Supplementary Materials and Methods**

**Behavioral evaluations.** Behavioral experiments were conducted on animals randomly assigned by experimenters blinded to the injection group.

***Actimetry****.* Mice were individually placed at the center of an infrared Actimeter apparatus (45x45x35 cm, BIOSEB; LE8816) composed by a 2-dimensional square frame, enlightened by a flood light (40 lux) placed above. Mice had 10 min exploration-time and their spontaneous behavior was tracked with velocity and distance moved recorded by Actitrack software (BIOSEB).

***Elevated Plus Maze****.* Anxiety-related behavior was assessed using an Elevated Plus Maze which is a cross platform with 2 open arms and 2 closed arms (80 cm long x 7 cm wide), enlightened by a flood light (open arms: 170-180 lux; closed arms: 50-60 lux) placed above. Mice were placed at the center of the maze with their face in the direction of a closed arm and were allowed to explore freely for 5 min. Time spent in each arm was recorded using the Ethovision XT tracking system (Noldus).

***Y-Maze.*** Short-term spatial memory was assessed in a spontaneous novelty-based spatial preference Y-maze test, composed of 3 arms (30 cm long x 8 cm wide), lit by a flood light (7-8 lux), placed above. Different extra-maze cues were placed on the surrounding walls. During the learning phase, mice were placed at the end of the “start” arm and were allowed to explore the “start” and the “familiar” arms for 5 min. Access to the third arm of the maze (“novel” arm) was blocked by an opaque door. The mouse was then removed from the maze and returned to its home cage for 2 min. In the test phase, the mouse was placed again in the “start” arm of the maze, the door of the “novel” arm was removed and the mouse was allowed to explore the maze for 1 min (**Figure 6A**). Allocation of the “novel” arm was randomly counterbalanced within each group. The amount of time the mouse spent in each arm of the maze was recorded during both learning and test phases using EthovisionXT (Noldus). For the learning phase, we calculated the percentage of time spent in the “familiar” arm vs. the “start” arm. For the test phase, a discrimination index as [novel arm/(novel+familiar arm)] × 100 was calculated.

***Barnes Maze****.* Spatial learning and long-term memory were evaluated using the Barnes maze task. This maze is a white circular PVC open platform surface (120 cm diameter) with 40 equally spaced holes (5 cm of diameter) located at 5 cm from its circumference and a black escape box (5x5 cm) located under one of the 40 holes. The platform is placed on a swivel system (to rotate it easily) in the center of the room, elevated 80 cm above the floor, lit by a flood light (800 lux) placed above and surrounded by spatial cues. Mice completed a habituation trial (5 min) to become familiar with the maze environment. Then, mice completed 4 days of acquisition training (learning phase) with 4 trials per day. For each trial, mice were placed in a start tube in the center of the maze (for 5-10 s) and then trained to locate the escape hole (randomized for all mice) using spatial cues surrounding the maze. If the mouse did not enter the escape hole in 3 min it was gently guided to the escape hole. Mice remained 60 s in the escape box before returning to its home cage. The inter-trial interval was 15 min. To reduce intra-maze odor cues, the maze surface and escape box were cleaned with 70% ethanol between each trial and the maze was rotated clockwise a quarter turn every day. Twenty-four hours after the last day of the training, mice completed a 2 min probe trial (test phase) where the escape box was removed. For the acquisition phase, the number of primary errors (number of times the mouse explores the other holes before finding the target one), primary latency (the time spent before finding the target hole) and distance moved (the distance travelled before finding the target hole) were recorded (**Figure 6C**). For the test phase, the time spent in the target quadrant was calculated.

**RNAseq*.*** mRNAs from astrocyte-enriched suspensions were extracted as described previously^39^. Full length cDNAs were generated from 500 pg to 1ng of total RNA using SMART-SeqX v4 UltraX Low Input RNA Kit for Sequencing (Takara Bio Europe, Saint Germain en Laye, France) according to manufacturer's instructions with 12 cycles of PCR for cDNA amplification by Seq-Amp polymerase. 600 pg of pre-amplified cDNA were then used as input for Tn5 transposon tagmentation by the Nextera XT DNA Library Preparation Kit (96 samples) (Illumina, San Diego, USA) followed by 12 cycles of library amplification. Following purification with SPRIselect beads (Beckman-Coulter, Villepinte, France), the size and concentration of libraries were assessed by capillary electrophoreris. Sequencing was performed on the GenomEast platform (Strasbourg) with 1x50 bp on a HiSeq 4000 System (Illumina). Reads were preprocessed in order to remove adapter, polyA and low-quality sequences (Phred quality score below 20) using cutadapt^40^ v1.10. After this preprocessing, reads shorter than 40 bases were discarded for further analysis. Reads were mapped onto the mm10 assembly of the Mus musculus genome using STAR^41^ v2.5.3. Gene expression quantification was performed from uniquely aligned reads using htseq-count^42^ v0.6.1p1, with annotations from Ensembl v102 and “union” mode. Only non-ambiguously assigned reads have been retained for further analyses. Read counts have been normalized across samples with the median-of-ratios method proposed by Anders and Huber^43^, to make these counts comparable between samples. Sequencing data that support the findings of this study have been deposited in the NCBI’s Gene Expression Omnibus (GEO) database (GSE272248). Comparisons of interest were performed using the test for differential expression proposed by Love et al.^44^ and implemented in the Bioconductor package DESeq2 v1.16.1. P-values were adjusted for multiple testing using the Benjamini and Hochberg method^45^. A functional annotation of deregulated genes was performed to identify the impacted biological processes, using DAVID (https://david.ncifcrf.gov/home.jsp). GSEA v4.1.0^46,47^ was also used as a pre-ranked analysis using the following settings “No collapsing of Gene Symbols, the classic Enrichment statistic, gene sets containing more than 500 genes and less than 50 genes were excluded from analysis” and with gene sets from GO, Pathway and MsigDB. Genes were ranked based on the values computed as such: -10*log10(pvalue) x fold change sense. Z-score expression for violin plot representation of RNA-seq data from the different modules was created with the R software.

**Immunohistological and analysis procedures.**

***Immunohistochemistry.*** 35 μm mouse floating sections were washed and permeabilized 3 times with phosphate buffer solution and 0.5% Triton X-100 (PBS-T). After an incubation with 0.3% H_2_0_2_ solution for 30 min to prevent the action of endogenous peroxidases, and the saturation of the tissue (5% of serum in PBS), the primary antibodies of A_2A_R, GFP, GFAP, Iba1 and c-Fos were incubated at 4°C (Serum, antibodies and incubation time are given in **Supplementary Table 2**). Biotinylated-conjugated secondary antibodies (1/500, Vector Laboratories) were then incubated for 1 h at RT and the revelation was performed using ABC method (#PK6100; Vector Laboratories) with 3,30-diaminobenzidine as the peroxidase substrate (#D9015; Sigma). The sections were mounted and incubated in successive ethanol baths (from 30% to 100%; 3x10 min) and toluene baths (3x10 min) once dried. Then the sections were covered with the Vectamount mounting medium (#H5000; Vector Laboratories) and coverslips (#LCO2460M; Labelians).

***Immunofluorescence.*** 35 μm mouse floating sections were washed and permeabilized 3 times with PBS-T. After the saturation of the tissue (5% of serum in PBS-T), the primary antibodies of A_2A_R, GFAP, S100β, Sox2, STAT3, Iba1, CD68, NeuN, YAP, HMGB1, RFP and GFP were incubated at 4°C (Serum, antibodies and incubation time are given in **Supplementary Table 2**). Alexa Fluor488-, Alexa Fluor568- and Alexa Fluor647-conjugated secondary antibodies (1/500; Life Technologies) were incubated for 1 h at RT. Sections were counterstained with DAPI (1/5000; #62248; Thermoscientific). The sections were mounted and treated for 10 min in 0.3% of Suden Black to block autofluorescence once dried. Then the sections were covered with Dako Fluorescent mounting medium (#S3023; Sigma) and coverslips (#LCO2460M; Labelians). For the STAT3 staining, we used a dedicated protocol requiring a permeabilization step thanks to a 100% methanol bath during 20 min at -20°C, before the saturation^49^.

***Image acquisition and analysis.*** For A_2A_R, GFP, GFAP, Iba1 and c-Fos immunohistochemistries, 6-7 sections per animal were acquired using x20 objective Zeiss Axioscan Z1 slide scanner. Quantification of the GFAP, Iba1 and C-Fos signal intensity and percentage of labeled area were performed using Fiji software (Java). For A_2A_R, GFAP, NeuN, Iba1, Sox2 and S100β immunostaining, images were acquired on a Zeiss LSM-710 confocal microscope taking-up to 15 z-stacks at 1.5 μm steps with a x63 oil objective (optimal frame size of 1348). For the CD68/Iba1, STAT3/YAP and HMGB1/S100β co-immunostainings, 3-4 regions of interest per animal were acquired on a Zeiss Spinning Disk high-resolution microscope taking-up to 15 z-stacks at 1.5 μm steps with a 40x oil objective. Percentage of labeled area of CD68^+^ in Iba1^+^ cells was performed using the Imaris plug-in “X-tension”. The density of microglia present in the CA1 stratum radiatum was determined by manually counting the number of Iba1^+^ microglia within a dedicated area using Imaris software. Signal intensity, percentage of labeled area and density of STAT3^+^, YAP^+^ or HMGB1^+^ cells in astrocytic cells (i.e, GFAP^+^ or S100β^+^ cells) as well as labeled area and volume of YAP^+^ and DAPI+ nucleus staining were performed using the Imaris plug-in “X- tension.

Signal intensity, percentage of labeled area and density of STAT3^+^ or YAP^+^ cells in GFAP^+^ cells as well as labeled area and volume of YAP^+^ and DAPI^+^ nucleus staining were performed using the Imaris plug-in “X-tension”. For the RFP/A_2A_R co-immunostaining, images were acquired on a Zeiss LSM-710 confocal microscope on 1 stack with a x40 oil objective using “mosaic” function in order to observe the astrocyte A_2A_R and neuronal hM3Dq expressions in the CA1 hippocampus area.

**Western Blots.** Protein concentrations of the samples above were quantified using the BCA assay (Pierce), diluted in lithium dodecyl sulphate buffer supplemented with reducing agents (Invitrogen) and then separated on 4-12% Criterion Bis-Tris Gels gels (Invitrogen). Proteins were transferred to nitrocellulose membranes, which were then saturated with 5% non-fat dried milk in TNT (Tris 15mM pH 8, NaCl 140mM, 0.05% Tween) and incubated at 4°C for 24h or 48 h with the primary antibodies. Appropriate HRP-conjugated secondary antibodies were incubated for 45 min at RT. Signals were visualized using chemiluminescence kits ECL (#RPN2106; Amersham) or ECL Prime (#RPN2232; Amersham) and an Amersham ImageQuant 800 imaging system. Quantiﬁcations were performed using ImageJ software and results normalized to β-actin. Primary and secondary antibodies used in this study are given in **Supplementary Table 2**. Uncropped gels are provided as **Supplementary material**.
