# Supplementary material for: Upregulation of adenosine A2A receptor in astrocytes is sufficient to trigger hippocampal multicellular dysfunctions and memory deficits
Source: Mol Psychiatry. 2025 Jul 23;30(11):5300–14. doi: 10.1038/s41380-025-03115-9 (PMC12532706; doi:10.1038/s41380-025-03115-9)
Supplement: Supplementary file 2 — Supplmentary figures [file 41380_2025_3115_MOESM2_ESM.pdf]

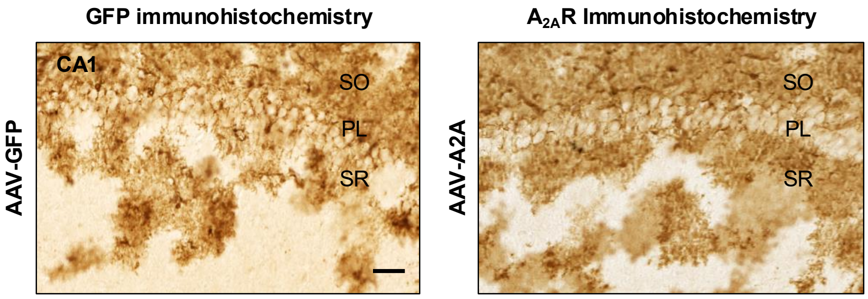

Supplementary Figure 1. AAV-based astrocytic A_{2A}R upregulation in CA1 hippocampus in mice. Representative images of GFP (left panel) or A_{2A}R (right panel) immunostainings highlighting GFP or A_{2A}R astrocytic upregulation in the *stratum oriens* (SO), pyramidal layer (PL) and *stratum radiatum* (SR) sublayers of CA1 hippocampus (scale bar=60 µm).

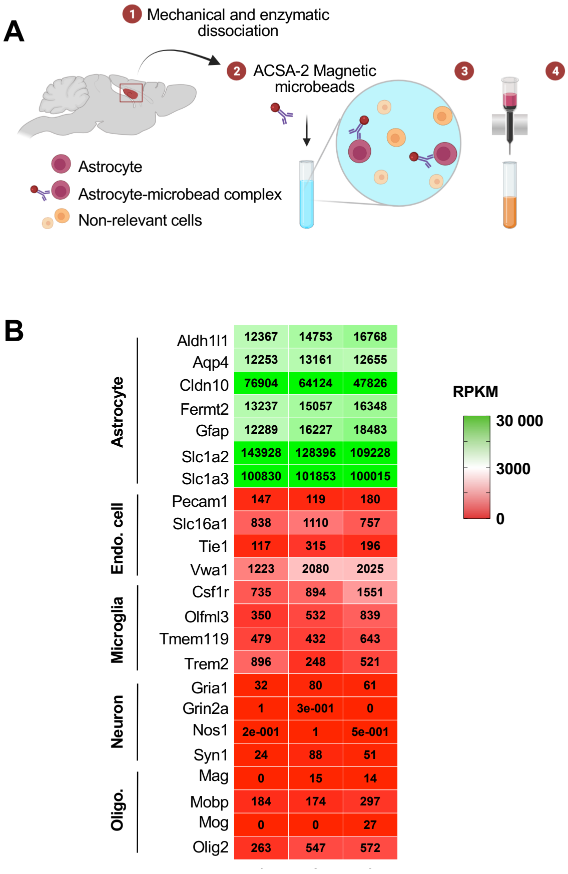

Supplementary Figure 2. Enrichment of hippocampal astrocytes using a magnetic-activated cell sorting method. (A) After cell dissociation of hippocampus (1), anti-ACSA-2 (Astrocyte Cell Surface Antigen) microbeads formed complexes with astrocytic cells (2). The magnetically labeled ACSA-2 cells (purple) are retained within a MS column thanks to a magnet -while the unlabeled cells (yellow) run through- and are eluted before RNA-sequencing (3-4). **(B)** Heatmap representing the enrichment (RPKM values) of several astrocytic genes vs. endothelial, microglial, neuronal and oligodendrocytic ones, attesting the reliability of the ACSA-2-based enrichment procedure.

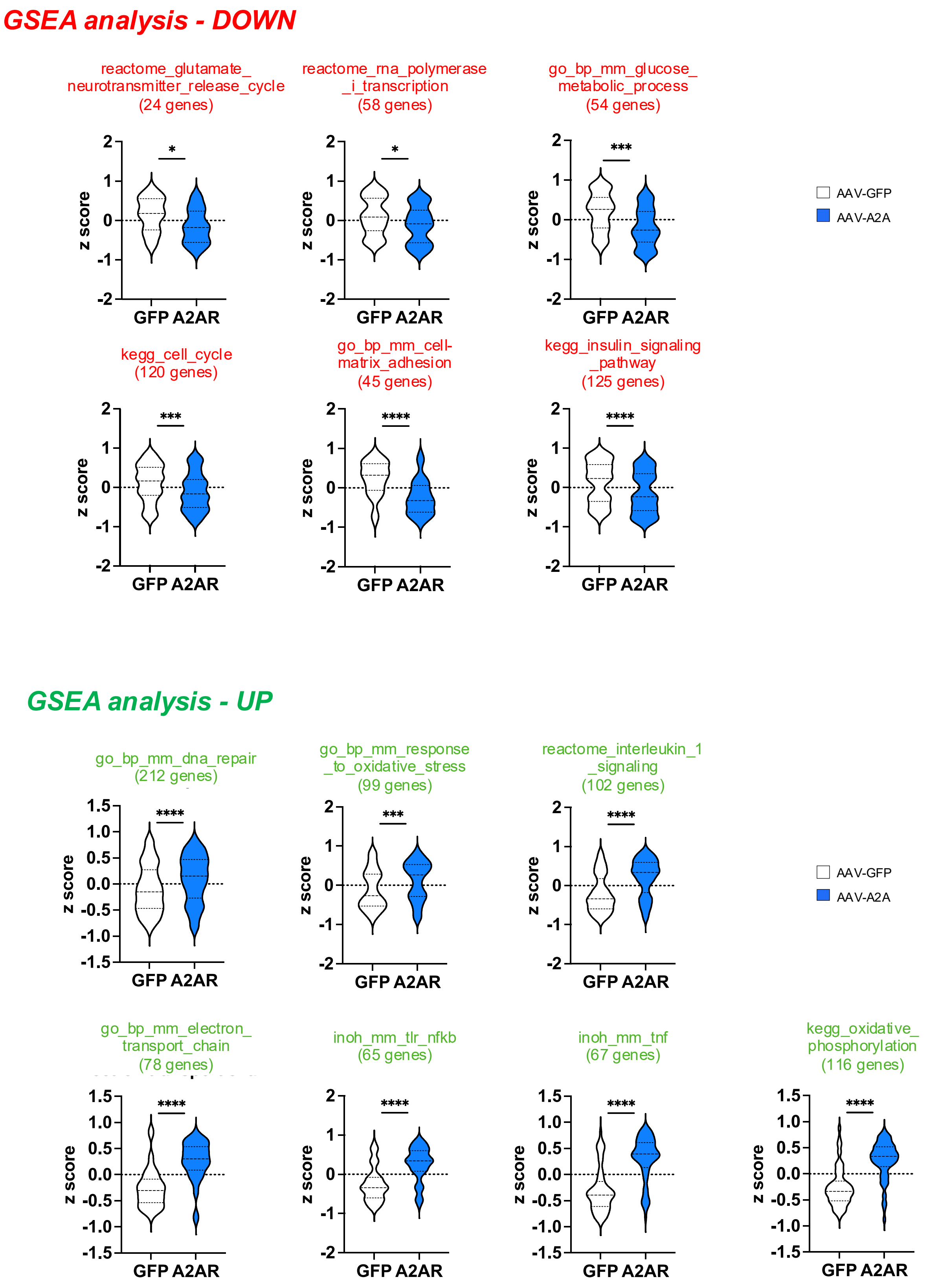

Supplementary Figure 3. Violin plots representing z-score expression values of genes belonging to the representative significant GSEA pathways shown on Figure 3. (N=3 animals per group). *P<0.05, **P<0.01, ***P<0.001, ****P<0.0001 using the non-parametric Mann-Whitney test.

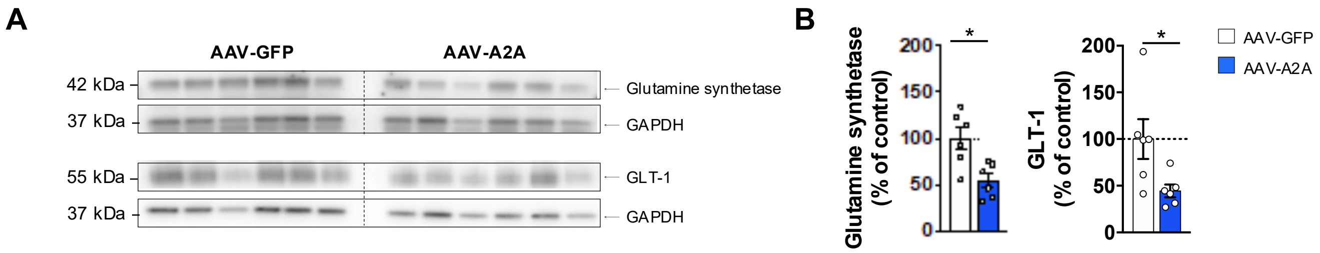

Supplementary Figure 4. (A) Glutamine synthetase (GS) and GLT-1 protein levels in the hippocampus of AAV-GFP and AAV-A2A mice. **(B)** Quantification shows a significant decrease of GS and GLT-1 in AAV-A2A vs. AAV-GFP mice (*P<0.05 vs. AAV-GFP; Student's t-test; N=6/group).

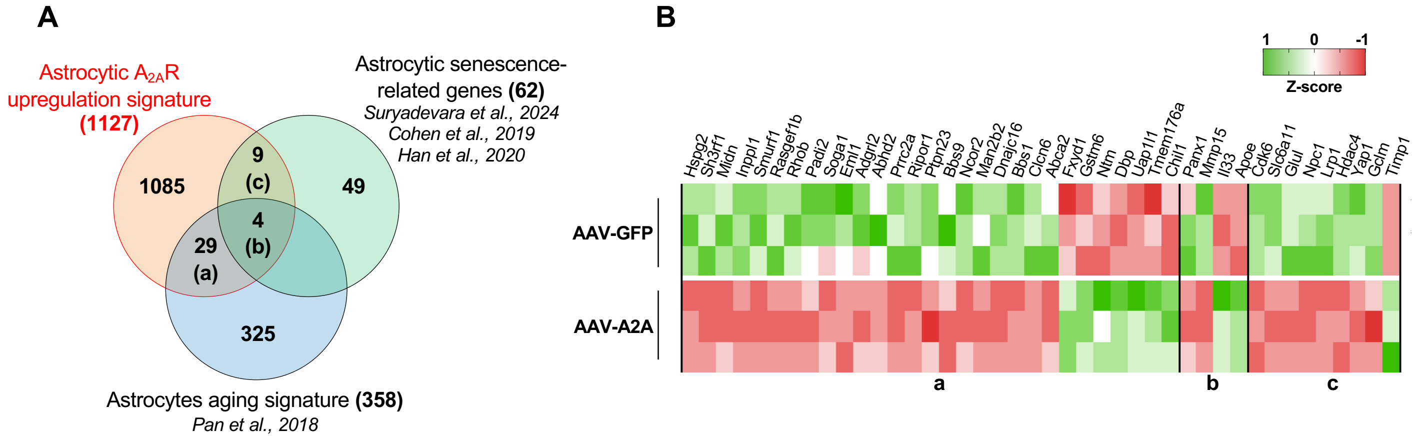

Supplementary Figure 5. (A) Venn diagram comparing the signatures of A_{2A}R-overexpressing astrocytes with signature of aged astrocytes (Pan et al., 2018) and astrocytic senescence-related genes. Common genes are annotated as a, b and c. **(B)** Heatmap representing the z-score expression (from RNA-Seq data) of common genes (a, b, c) uncovered by the Venn diagram.

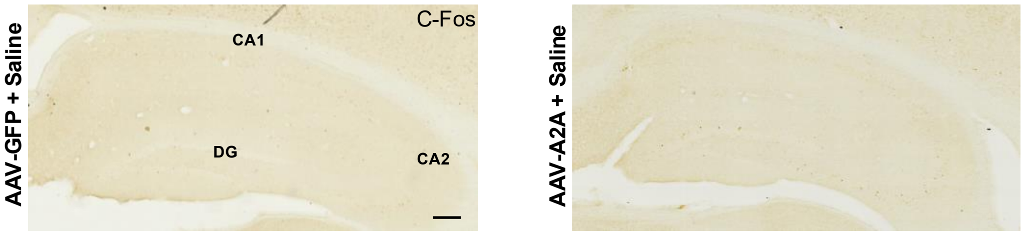

Supplementary Figure 6. Representative images of c-Fos immunostaining in the CA1, CA2 and DG regions of hippocampus in AAV-GFP (left panel) and AAV-A2A (right panel) animals treated with saline (scale bar=400 µm; N=4-5 mice/group).

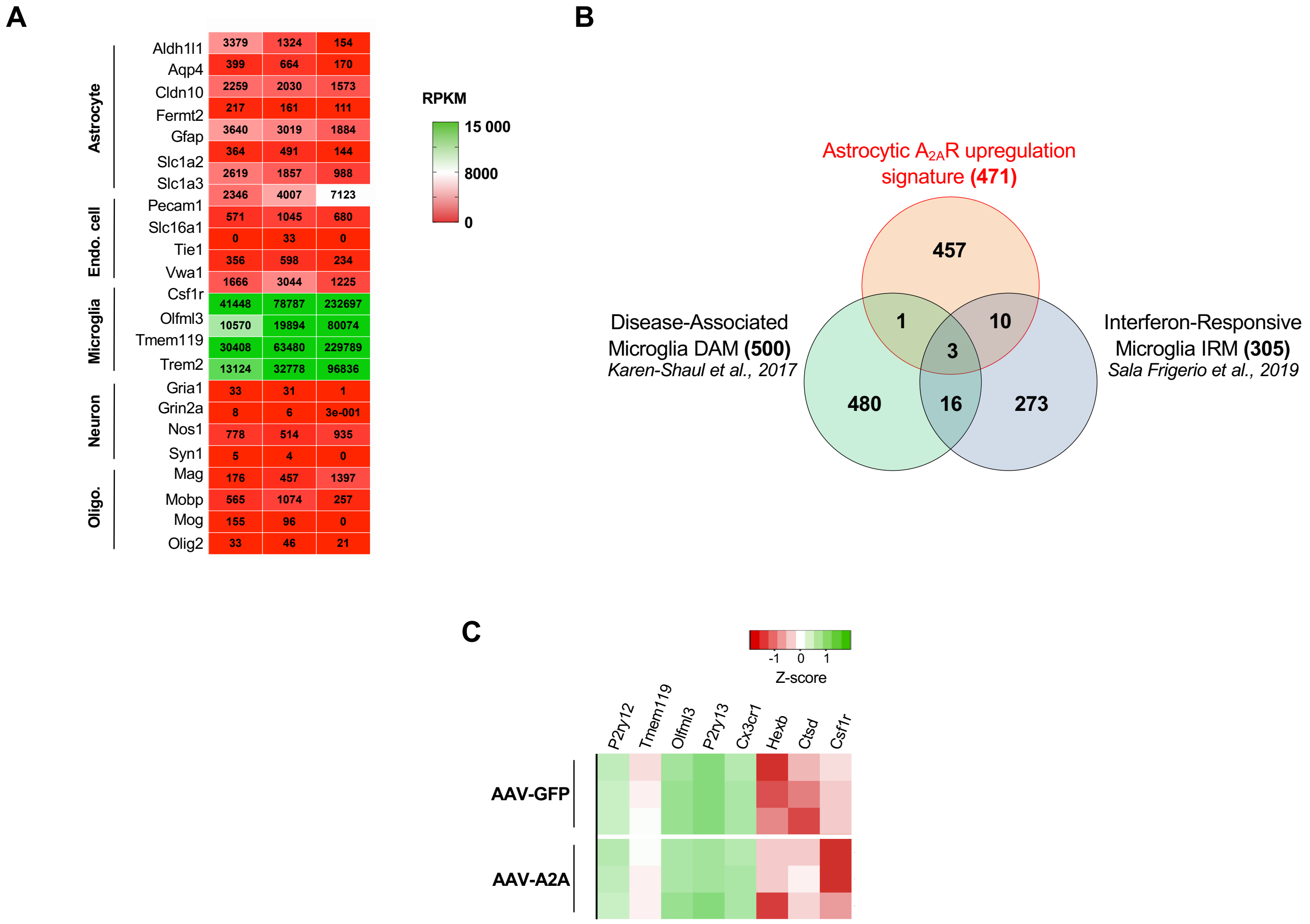

Supplementary Figure 7. Microglial transcriptomic signature following astrocytic $A_{2A}R$ upregulation in hippocampus vs GFP control. **(A)** Heatmap representing the enrichment (RPKM values) of several microglial genes vs. astrocytic, endothelial, neuronal and oligodendrocytic ones, attesting the reliability of the CD11b-based enrichment procedure. **(B)** Venn diagram comparing the microglial signature of $A_{2A}R$-overexpressing astrocytes to signatures of Diseases-Associated Microglia (DAM; Keren-Shaul et al., 2017) and Interferon-Responsive Microglia (IRM; Sala Frigerio et al., 2019). **(C)** Heatmap representing the z-score expression (from RNA-Seq data) of common homeostatic microglial genes and showing no difference between groups.

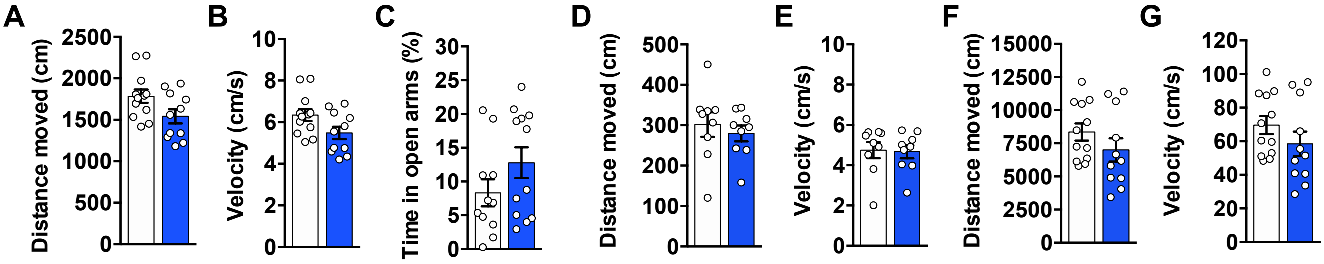

Supplementary Figure 8. Locomotor activity and anxiety behavior. (A-C) During the Elevated-Plus maze, no difference regarding the distance moved **(A)**, the velocity **(B)** as well as the time spent in the open arms **(C)** was observed between the AAV-A2A and AAV-GFP animals (N=11-12 mice/group; P>0.05; Student's t-test). During the Y-maze test **(D,E)**, no difference regarding the distance moved **(D)** and the velocity **(E)** was observed between the AAV-A2A and AAV-GFP animals (N=9 mice/group; P>0.05: Student's t-test). **(F,G)** Same applied in the Barnes task (N=11-12 mice/group; P>0.05; Student's t-test).
