# Supplementary figures and images for: Upregulation of adenosine A2A receptor in astrocytes is sufficient to trigger hippocampal multicellular dysfunctions and memory deficits
Source: Mol Psychiatry. 2025 Jul 23;30(11):5300–14. doi: 10.1038/s41380-025-03115-9 (PMC12532706; doi:10.1038/s41380-025-03115-9)

### Membrane #113

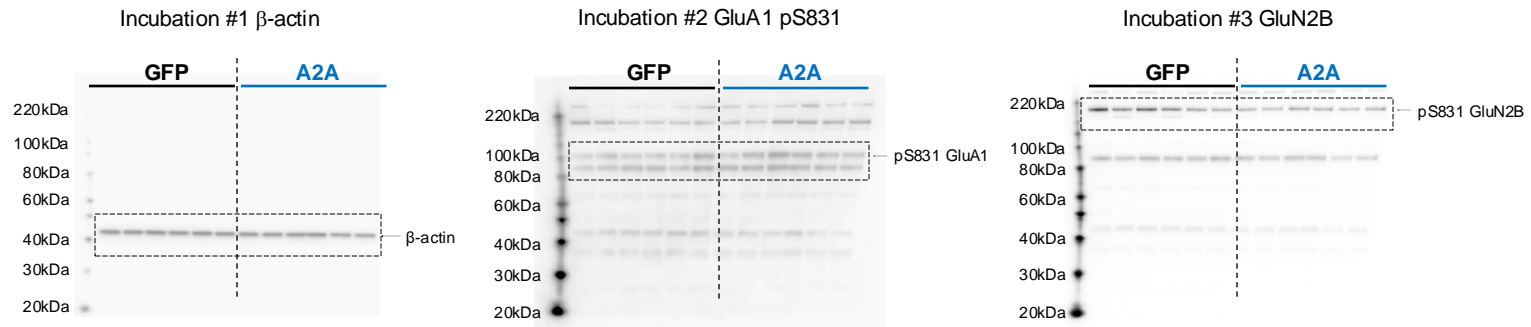

### Membrane #201

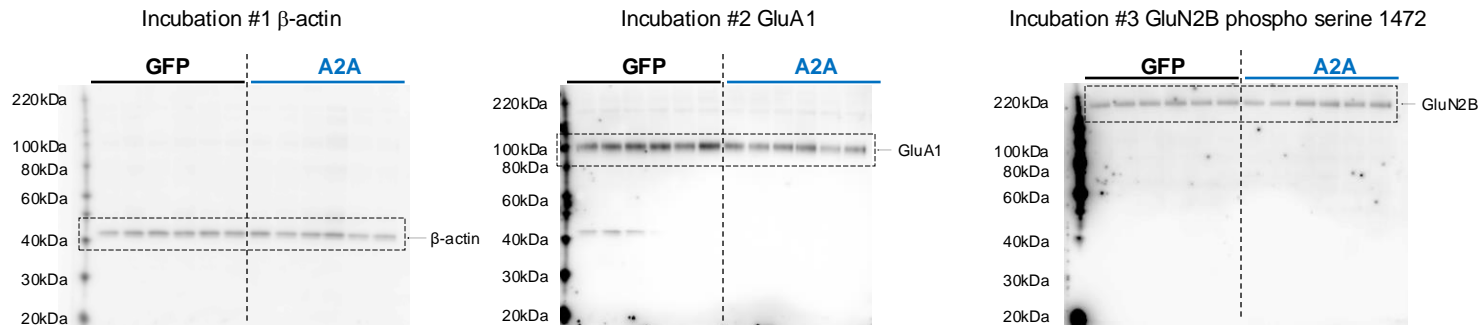

### Membrane #70

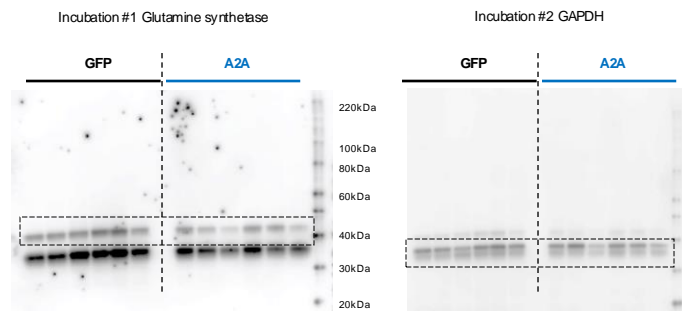

### Membrane #73

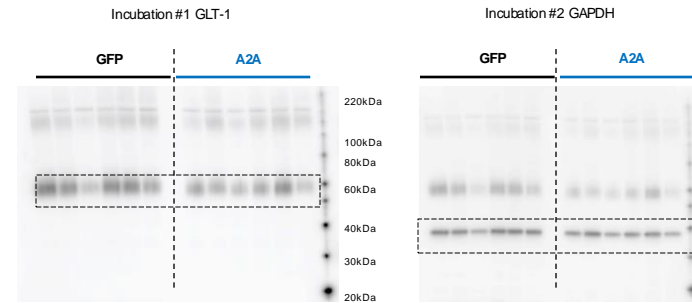

Supplement: Supplementary file 3 — Supplementary data - uncropped gels [file 41380_2025_3115_MOESM3_ESM.pdf]
